# Exploring the potential of a structural alphabet-based tool for mining multiple target conformations and target flexibility insight

**DOI:** 10.1371/journal.pone.0182972

**Published:** 2017-08-17

**Authors:** Leslie Regad, Jean-Baptiste Chéron, Dhoha Triki, Caroline Senac, Delphine Flatters, Anne-Claude Camproux

**Affiliations:** 1 Molécules thérapeutiques in silico (MTi), INSERM UMR-S973, Paris, France; 2 Université Paris Diderot, Sorbonne Paris Cité, Paris, France; 3 Institut de Chimie de Nice, UMR-CNRS 7272, Faculté des Sciences, Université de Nice-Sophia Antipolis, Nice, France; 4 Sorbonne Universités, UPMC Univ Paris 06, CNRS, INSERM, Laboratoire d'Imagerie Biomédicale (LIB), Paris, France; University of Queensland, AUSTRALIA

## Abstract

Protein flexibility is often implied in binding with different partners and is essential for protein function. The growing number of macromolecular structures in the Protein Data Bank entries and their redundancy has become a major source of structural knowledge of the protein universe. The analysis of structural variability through available redundant structures of a target, called multiple target conformations (MTC), obtained using experimental or modeling methods and under different biological conditions or different sources is one way to explore protein flexibility. This analysis is essential to improve the understanding of various mechanisms associated with protein target function and flexibility. In this study, we explored structural variability of three biological targets by analyzing different MTC sets associated with these targets. To facilitate the study of these MTC sets, we have developed an efficient tool, SA-conf, dedicated to capturing and linking the amino acid and local structure variability and analyzing the target structural variability space. The advantage of SA-conf is that it could be applied to divers sets composed of MTCs available in the PDB obtained using NMR and crystallography or homology models. This tool could also be applied to analyze MTC sets obtained by dynamics approaches. Our results showed that SA-conf tool is effective to quantify the structural variability of a MTC set and to localize the structural variable positions and regions of the target. By selecting adapted MTC subsets and comparing their variability detected by SA-conf, we highlighted different sources of target flexibility such as induced by binding partner, by mutation and intrinsic flexibility. Our results support the interest to mine available structures associated with a target using to offer valuable insight into target flexibility and interaction mechanisms. The SA-conf executable script, with a set of pre-compiled binaries are available at http://www.mti.univ-paris-diderot.fr/recherche/plateformes/logiciels.

## Introduction

Proteins are highly flexible macromolecules and their dynamic properties are crucial to many biological processes. This flexibility is often implied in interactions between different partners and is essential for protein function, e.g., enzyme catalysis and activity regulation [1–3]. The analysis of the protein flexibility is relevant to decipher the variety of mechanisms associated with a function (e.g., allostery, aggregation, and oligomerization) and protein plasticity, as described in [4,5]. The growth in the speed of macromolecular structure determination techniques has increased the number of Protein Data Bank (PDB) [6] entries to approximately 122,000 three-dimensional (3D) protein structures. Because most newly solved structures fall within existing families, a large number of structures exhibit high redundancy, i.e., more than half share at least 95% sequence identity. Even if this redundancy is considered valuable as in investigating families of homologous sequences [7,8], the dominant approach for the data mining of the PDB considers redundancy as non-informative [9], resulting in an artificial reduction in the variability of the structural space. The different structures associated with a target, referred as multiple target conformations (MTC), can correspond to available PDB structures obtained under different conditions using nuclear magnetic resonance (NMR) models and X-ray crystallography or to theoretical models obtained using homology modeling or to dynamic series of 3D views obtained using molecular dynamics simulations that sample exhaustively the target conformation landscape. These ensembles of 3D view of one target correspond to different MTC subsets when obtained by different sources and approaches.

A structural analysis and comparison of MTC sets associated with a target allow to investigate structural variability and to capture information about its flexibility. The analysis of MTCs associated with subtle variations in their amino-acid (AA) sequences or corresponding to multiple sequences with the same fold, can highlight close sequence-structure relationships [10]. The analysis of an MTC set associated with different structural elucidation methods or biological contexts (i.e., forms, conditions of pH or temperature) can distinguish structural variability due to different experimental conditions. The analysis of a homogeneous MTC set without partner or conditions changes can locate the intrinsic structural flexibility of the target. The analysis of a heterogeneous MTC set including free and bound conformation forms can highlight structural variability induced by a partner (protein, nucleic acid or ligand) binding, corresponding to induced-fit effects.

Currently, the tools dedicated to the target variability analysis are based either on the MTC sequence or on structural variability analysis. Available tools dedicated to MTC sequence analysis (comparison sequence or multiple sequence alignment (MSA) computation), such as ConSurf [11] and MatrixPlot [12], are applied to identify conserved or mutated residues in a sequence set. Multiple structural alignment tools, such as MUSTANG [13] and MulPBA programs [14], are proposed to compare globally 3D structures. RMSD (root-mean-square-deviation) metrics can also be computed between all 3D structure superimposed pairs [15,16] or between MTC superimposed in a same reference (which has to be identified among MTC) [17]. Some recent tools, CoDNaS [16,18] and PSSweb server [19,20] focus on the quantification of MTC local structural variability. CoDNaS analyzes the target structural diversity by computing pairwise residue RMSD between available PDB structures with more than 95% of sequence identity with the target. PSSweb server provides local structural statistics of a MTC set by computing the standard deviation of atom coordinates. Both these tools provide useful information on the target local structural variability but not directly coupled with the sequence variability information. Other methods propose to analyze the structural variability of a target using a structural alphabet (SA). An SA proposes a systematic decomposition of protein 3D structures into finite sets of generic short fragment prototypes labeled by structural letters (SLs) [21–25]. An SA reduces the 3D conformational complexity by simplifying any 3D conformation in a series of SLs. Then it simplifies the comparison of 3D conformations, encoding into SL sequences, in the well-known comparison of sequences. SA have been exploited in the past for a number of applications, including local structure flexibility prediction [26], structure mining [27–29], to classify protein fold [30]. SA approach is therefore particularly adapted to compare and characterize of structural variability [31] and to characterize and predict protein flexibility [32]. A tool based on a SA of 28 SLs, called “GSATools” was developed to analyze an ensemble of molecular dynamics models associated with the same sequence [23] and combined to molecular simulation to increase the exploration of the conformational space of proteins [33]. Based on another SA of 16 SLs, Mahajan et al. (2014) compared the local conformation variations observed at structurally equivalent positions of a multiple structural alignment obtained using NMR models and different homologous structures of a single protein [34]. These last two SA-based approaches have been successfully applied to local conformation variability analysis but do not provide information about sequence-structure variability relationship analysis and have not been adapted for the analysis of various and heterogeneous MTC sets.

In this paper, we propose to explore the potential of a SA-based approach for target flexibility insight, taking into account both sequence and structural variability. To this aim, we have developed a new tool, referred to as SA-conf, using our previously developed hidden Markov model-SA (HMM-SA) of 27 SLs [24,35,36]. We chose HMM-SA because it provides a very precise description of protein structures, particularly of loop regions [37,38], it was demonstrated relevant to explore the local backbone deformation involved in protein-protein interactions [39,40]) and to generate 3D peptide conformations [41,42]. SA-conf is able to perform variability analysis of a MTC set at three levels of protein description: sequence, secondary structure and 3D structure. Moreover, it allows the analysis of all structure types obtained using different methods such as experimental methods (X-ray crystallography, NMR) and theoretical modeling. SA-conf mines and produces an overview of any considered MTCs in terms of the sequence and local structural compositions and associated experimental conditions. Then, it provides joint sequence and local structural variability quantification using Shanon entropy criteria from a common MTC alignment. The relevance of MTC sets variability analysis with our SA-conf tool to capture flexibility information is illustrated on three biological targets of interest and well studied: the human urokinase-type plasminogen activator (uPA), the p53 DNA-binding domain (DBD) and the protease of the immunodeficiency virus type 1 (PR1). Our results confirm that taking into account and coupling different MTC obtained by NMR, X-ray crystallography and, homology modeling, allow capturing precisely target variability and offers clues for flexibility interpretation by identifying the different sources of target flexibility such as induced by partner binding, by mutations and intrinsic flexibility in agreement with literature.

## Materials and methods

### Presentation of three biological targets

The three targets (uPA, p53 and PR1) analyzed in this work are intensively studied in the literature and are known to bind to different partners. Moreover, a large number of structures are available in the PDB for those proteins, corresponding to different target forms (free and bound structures, wild-type and mutant structures).

We built different MTC subsets extracted from the PDB for each target to identify structural variability induced by mutations, partner binding (DNA, protein or ligand), explained by intrinsic flexibility or by different protein structure determination methods such as NMR, X-ray crystallography. In addition, we explored the variability from a set of p53 models obtained by homology modeling.

### The uPA catalytic domain target

The uPA transforms plasminogen to plasmin, a protease with broad specificity that activates matrix metalloproteases. It is a 411-residue protein, consisting of three domains: the serine protease catalytic domain, the kringle domain and the growth factor domain. The uPA plays an essential role in the process of tumor cell migration and metastasis, aortic aneurysm, and multiple sclerosis [43]. Metastatic cancer cells are marked by uPA overexpression [44,45]. Thus, it is an attractive therapeutic target. One strategy for diminishing the uPA activity is to develop inhibitor(s) that directly bind and influence the catalytic activity of uPA. In this work, we studied the flexibility of the uPA target upon the inhibitor binding using our SA-conf tool. In a first step, SA-conf was used to build an MTC set, “uPA set”, from the PDB in which conformations are selected from different criteria (Table 1). We extracted the inhibitor-binding site of the uPA catalytic domain complexed with an inhibitor (PDB ID 3I6G) using PockDrug-Server [46] to analyze the uPA catalytic domain structural variability linked to the inhibitor binding.

**Table 1:** Description of the subsets used to test SA-conf.

Set Name		uPA	PR1	PR1-NMR	P53	P53-NMR	P53-HM_QM_	P53-HM_HM_
Size		105	33	28	78	36	100	100
Heterogeneous		x	x		x			
Homogeneous				x		x	x	x
Structure determination methods	X-ray	x	x (31)		x			
	NMR		X (2 –first models of PDB ID 1BVE and 1BVG)	x	x	x		
	Homology models			x			x	x
Complexed with a partner (protein/nucleic acid)	Free form	x			x	x		
	Bound form		x	x	x		x	x
Complexed with a ligand	Apo form	x			x	x	x	x
	Holo form	x	x	x	x			
Sequence type	Wild-type	x	x		x	x	x	x
	Mutants	x	x	x	x			
Supplementary information			Chain A of HIV protease (isolate HXB2 –UniProt ID: P04585)	NMR models are extracted from file corresponding to the PDB ID 1BVE		NMR models are extracted from file corresponding to the PDB ID 2FEJ	Wild-type models were generated using Modeler software (Sali & Blundell, 1993)	
							Template: p53 quadruple mutant (QM, PDB ID: 1UOL)	Template: p53 hexamutant (HM, PDB ID: 2WGX)

#### The p53 DBD target

The p53 protein exerts a tumor suppressor function primarily as a transcription factor by regulating the expression of a set of genes in the cell. More than 50% of human cancers are associated with mutations in p53 and 90% of them occur in the DBD. The human p53 is a 393-residue protein that can be structurally and functionally divided into four domains. The p53 DBD is located in the central region of the protein and contains approximately 200 residues. Its conformation consists of a β-sandwich composed of two antiparallel β-sheets with two large loops referred to as L2 and L3. A loop (L1)-sheet-helix (H2) motif (LSH motif) and the L3 loop are involved in direct DNA interactions [47]. p53 DBD is able to bind to other proteins, such as 53BP1, 53BP2 or BCL-XL and small molecules have recently been reported to rescue the p53 DBD mutant [48–50].

To analyze the structural flexibility of the p53 DBD associated with mutations, different structure resolution methods (experimental or theoretical), induced-fit partner binding (DNA, protein or ligand) or explained by intrinsic flexibility, we built four subsets of p53 DBD (Table 1). First, a heterogeneous MTC subset, “P53 set”, was built using SA-conf tool, it is composed of divers forms of p53 DBD structures. A second homogeneous subset, “P53-NMR”, contained the 36 NMR models of a wild-type human unbound p53 DBD (S1 Fig, Table 2). In parallel, two additional subsets were built to analyze the structural variability associated with a set of homology models obtained using the MODELLER 9 program [51]. These subsets, “P53-HM_QM_” and “P53-HM_HM_”, are composed of wild-type models built by homology modeling using two mutated p53 DBD X-ray structures as templates: one super stable quadruple mutant (QM) and one hexamutant (HM) structure. To analyze the structural variability of p53 protein linked to inhibitor binding, we extracted its inhibitor-binding site from a holo p53 structure (PDB ID 4AGM) using PockDrug-Server [46].

**Table 2:** Sequence and structural quantification of the three datasets analyzed using the SA-conf tool.

	MTC sets							
		uPA	P53	P53-NMR	P53-HM_QM_	P53-HM_QM_	PR1	PR1-NMR
	Number of positions	261	241	204	195	195	99	99
All positions	Average *neq*_*AA*_ ± sd	1.02 ± 0.17	1.1± 0.22	-	-	-	1.09 ± 0.27	-
	*neq*_*AA*_ maximum value	2.86	2.57	-	-	-	3.06	-
	Average *neq*_*SL*_ ± sd	1.66 ± 0.75	1.81± 0.95	2.69± 2.16	1.24 ± 0.41	1.25 ± 0.39	1.89 ± 0.85	1.99 ± 1.04
	*neq*_*SL*_ maximum value	5.08	5.58	12.76	3.14	2.40	4.47	6.23
	Number of conserved positions in terms of AA (*neq*_*AA*_ = 1)	98% (240)	76% (149)	-	-	-	84.8% (84)	-
	Number of structurally conserved positions (*neq*_*SL*_ = 1)	9% (22)	14% (27)	9.5% (19)	62% (119)	54.7% (105)	12.5% (12)	24% (23)
	Number variable regions	9	8	7	8	9	8	8
Structurally variable regions	Number of positions (%)	57 (22%)	52 (22%)	115 (56%)	23 (11%)	28 (14%)	43 (43%)	44 (44%)
	Average *neq*_*AA*_ ± sd	1 ± 0.008	1.06 ± 0.13	-	-	-	1.11± 0.34	-
	Average *neq*_*SL*_ ± sd	2.48 ± 0.90	2.92 ± 1.05	3.43 ± 2.56	1.97 ± 0.46	1.95 ± 0.28	2.55 ± 0.83	2.66 ± 1.07

#### Immunodeficiency virus type 1 protease target

PR1 is an effective drug target for acquired immune deficiency syndrome treatment because it is involved in the maturation of the viral proteins [52]. PR1 is a homodimer composed of 99 amino acids in each chain. Its substrate-binding pocket is formed by residues from both subunits and it is located at the protein-protein interface. PR1 contains a flexible beta hairpin known as the “flap region”, that is crucial for the PR activity. Indeed, a large-scale flap opening is presumably required for normal substrate access to the active site. Thus, consistent structural differences are observed between the apo and holo forms of PR1, particularly at the level of flap regions, i.e., in closed form, pulled in toward the bottom of the active site in the holo form versus in a semi-open conformation in the apo form. To analyze the PR1 structural flexibility associated with the ligand binding and different structure resolution methods, we prepared two MTC subsets of the PR1 in holo form (Table 1). The first set is a heterogeneous one: “PR1 set”, composed of 33 chains A of PR1 complexed with different ligands. The second set is a homogeneous one: “PR1-NMR set”, composed of the 28 NMR models extracted from one PR1 mutant complexed with an inhibitor. We extracted the inhibitor-binding site of PR1 using PockDrug-Server [46] and the PR1 complexed with an inhibitor (PDB ID 1HXB).

### SA-conf tool protocol

**Fig 1:**
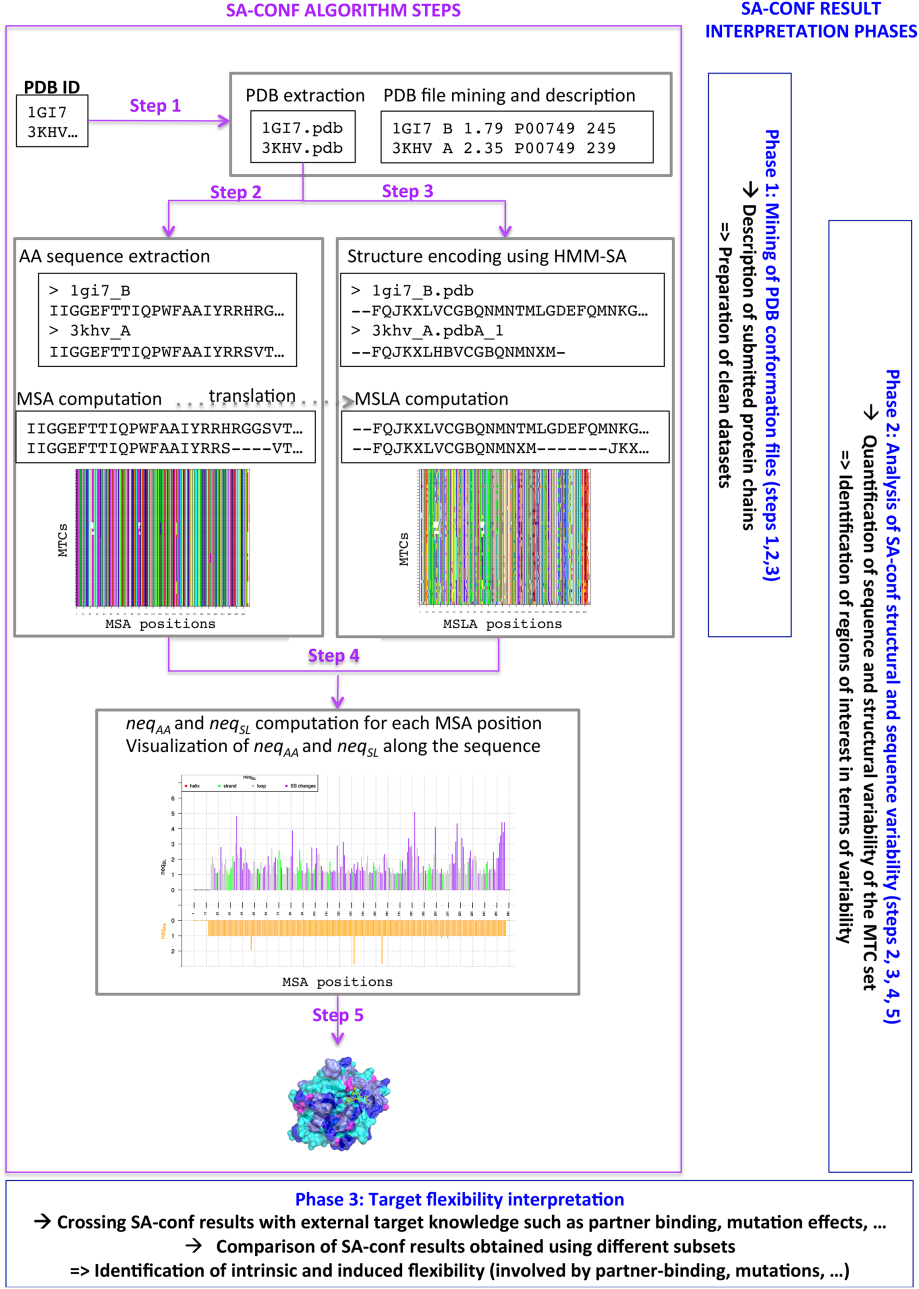
SA-conf algorithm description. The left part presents the different steps of the SA-conf algorithm. SA-conf starts from a list of MTC corresponding to PDB IDs. Step 1 corresponds to the extraction of the MTC information. Step 2 corresponds to the AA sequence extraction from all PDB files and the computation of the MSA. Step 3 corresponds to the extraction of the local conformations of all MTC residues, using the SLs of HMM-SA [24]. From the obtained SL sequences, SA-conf computes an MSA-derived structural alignment of the MTC (MSLA). Step 4 corresponds to the detection of MSA and MSLA variable positions and regions using exponent of Shanon entropy criteria (*neq*_*AA*_ and *neq*_*SL*_, respectively) and secondary structure changes. Step 5 corresponds to the localization of the significant positions in the target 3D structure. The right part of the figure presents the different phases of the variability analysis using SA-conf results.

In the framework of this study, we had to develop a tool, called SA-conf, for mining diverse MTC sets and extracting target flexibility information with no preliminary data preparation required. It is based on five different steps, as described in the following and detailed in Fig 1.

#### Step 1—Extraction of conformation information

One advantage of SA-conf is that it can work with 3D structures sets composed of PDB files or structures not available in the PDB, such as models generated using molecular dynamics simulation or homology modeling. In SA-conf, a structure can correspond to one (monomer), several chain(s) (oligomers, e.g., 1GI7) or a protein chain (e.g., 1GI7_B). To run SA-conf, the user submits a text file containing the structure ID list (PDB one or artificial one) where each ID must be in PDB format (4 characters) and a directory containing the structure files of ID not available in the PDB.

Then, SA-conf parses the structure files and creates a description of each file in terms of the experimental approach used; the associated resolution for X-ray structures or the number of models for NMR structures; the number of chain(s), their length(s) and their associated UniProt IDs; the names of the HETATM atoms, i.e., the atomic coordinate records used to describe the atoms presented in HET groups (atoms within "non-standard" groups present in the structure file).

#### Step 2—Sequence extraction and multiple sequence alignment computation

**Fig 2:**
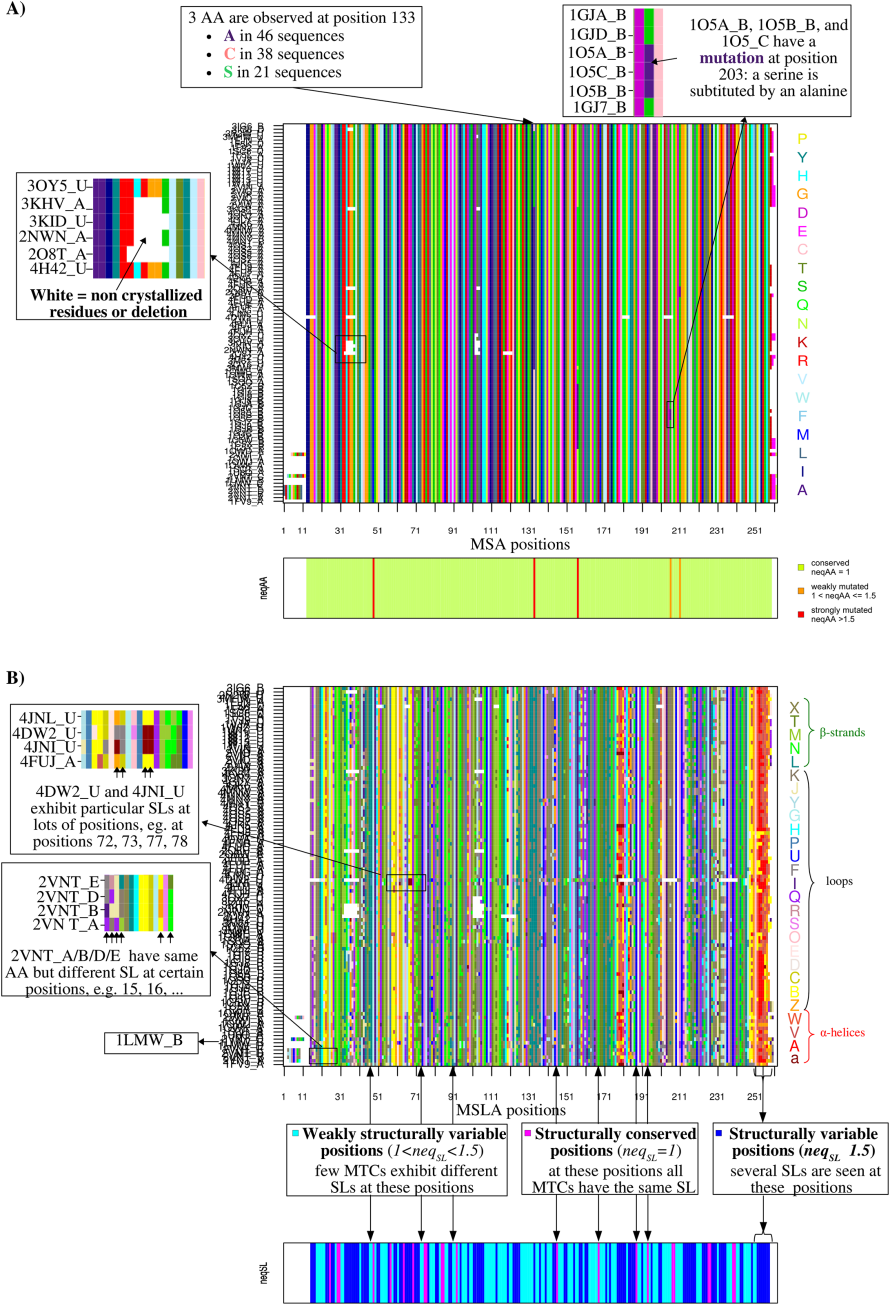
Visualization of the sequence and structural variability of the uPA set. (A) Amino-acid sequence variability of the uPA set. The upper figure presents the MSA map (SA-conf step 2 output named AA_alignment.pdf) obtained using the set of 184 human uPA catalytic domains and ClustalW program and manually curated. A total of 184 aligned AA sequences are presented in rows and the 261 MSA positions are shown in columns. Each position is colored according to the 20 AA types. The bottom figure presents sequence conservation/variability of each uPA set position in terms of *neq*_*AA*_. Positions are colored according to their *neq*_*AA*_ values. Sequence conserved positions, i.e., positions exhibiting a *neq*_*AA*_ value of 1, are colored in light green. Sequence weakly variable positions, i.e., positions exhibiting a *neq*_*AA*_ value included from 1 to 1.5, are colored in orange. Sequence strongly variable positions, i.e., positions exhibiting a *neq*_*AA*_ value larger than 1.5, are colored in red. (B) Structural variability of the uPA set. The upper figure presents the MSLA map (SA-conf step 3 output, named SL_alignment.pdf) computed using the 184 uPA catalytic domains. In the MSLA, the 184 aligned SL sequences are shown in rows and the 261 positions are shown in columns and colored according to the 27 SLs [24]. [a, A, V, W]-SLs primarily found in the α-helix are colored in red, and [L, M, N, T, X]-SLs primarily found in the β-strand are colored in green, other SLs correspond to loop [24]. The bottom figure presents structural conservation/variability of each uPA set position in terms of *neq*_*SL*_. Positions are colored according to their *neq*_*SL*_ values. Structurally conserved positions, i.e., positions exhibiting a *neq*_*SL*_ value of 1, are colored in magenta. Structurally weakly variable positions, i.e., positions exhibiting a *neq*_*SL*_ value included from 1 to 1.5, are colored in cyan. Structurally strongly variable positions, i.e., positions exhibiting a *neq*_*SL*_ value larger than 1.5, are colored in blue.

The amino acid (AA) sequences of the *C* protein chains are extracted from the input structures. An MSA of the *C* sequences is computed using the ClustalW [53] (by default) or T-coffee algorithms [54]. The choice of MSA algorithms depends on the similarity of the sequence set: T-coffee algorithms will be preferred for dissimilar sequences with large insertions/deletions [55,56]. The user can also submit a pre-computed MSA. In output, SA-conf produces a graphic that presents the MSA with the *C* aligned AA sequences in rows and the *p* corresponding multiple alignment positions in columns (Fig 2A). Each position is colored according to the 20 AA types. SA-conf also produces a table that contains the correspondence between the MSA position numbers, the PDB position numbers in *C* chains and the associated UniProt numbers if a UniProt ID list is submitted.

#### Step 3 –Multiple structural alignment based on simplified conformations

The local structural information of the *C* chains is extracted using the structural alphabet HMM-SA that we previously developed [24,35,36]. It is a classification of four-Cα fragments established by HMM using the fragment geometry similarity into 27 classes, named structural letters (SLs) and labeled [*a*, *A-Z*] (S2 Fig). HMM-SA is a very effective tool to describe the protein local conformations deeply with four SLs specific to α-helices, five SLs specific to β-strands and the 18 remaining SLs that finely encode loop conformations [24]. This SA-based definition of secondary structures exhibits a consensus of 83% [37] with STRIDE method [57]. HMM-SA has demonstrated its interest to accurately decompose the loop regions [37,38], to extract functional motifs [58,59], to characterize protein-protein interactions [60] and to explore the backbone deformation [39,40], to analyze side-chain conformations [27], to mine protein structure [28], to classify protein folds [30] and recently to predict peptide conformations [41,42].

During the SA-conf process, each protein chain of *p* residues is encoded using HMM-SA into a *(p-3)* SL sequence, where each SL describes the local geometry of each four-Cα fragment (*i*, *i + 1*, *i + 2*, *i + 3)* and is assigned to the third residue (*i + 2*) of the four-Cα fragment. To perform a direct comparison of the AA and SL variability of the *C* chains, SA-conf encodes the MSA into a multiple SL alignment (MSLA) by replacing each MSA residue with its corresponding SL. SA-conf produces in output a graphic that presents the MSLA, where the *C* aligned SL series are presented in rows and the (*p-3)* MSLA positions are presented in columns and colored according to the 27 SLs in agreement with the secondary structure three-state classification (Fig 2B). Thus, the *i*^th^ column of the MSLA indicates the *C* different SLs observed at position *i*. If several SLs are observed in a given position, this means that some chains exhibit different local conformations. This observed SL-change highlights local structural backbone deformation of the target in the corresponding positions.

#### Step 4—MSA and MSLA analysis in terms of variable positions

SA-conf determines the conservation and variability of the *C* chains using the three levels of protein description: AA sequence, secondary structures and 3D structures described by local structures. The exponent of the Shannon entropy [32] is used as a variability index to quantify the number of different AAs and SLs observed in each MSA or MSLA position. The exponent of the Shannon entropy parameters, noted respectively *neq*_*AA*_ and *neq*_*SL*_, quantify the amount of information delivered by a position *i* in terms of AAs and SLs, respectively and were computed according to Equation Equation 1

$$neq_{AA}(i)=\text{e}\text{x}{\text{p}}^{-\sum_{j=1}^{20}freq(aa_{j}^{i}).\text{ln}(freq(aa_{j}^{i}))}$$

$$neq_{SL}(i)=\text{e}\text{x}{\text{p}}^{-\sum_{j=1}^{27}freq(sl_{j}^{i})\text{ln}(freq(sl_{j}^{i}))}$$

where *freq(aa*_*j*_^*i*^) and *freq(sl*_*j*_^*i*^*)* are the frequencies of the *j*^*th*^ AA, named *aa*_*j*_, and the *j*^*th*^ SL, named *sl*_*j*_ observed at MSA or MSLA position *i*, respectively.

The exponent of the Shannon entropy takes into account both the average quantity of information of the position *i*, i.e., number of observed letters (AA or SL) at this position, and its uncertainty. The *neq*_*AA*_ values vary between 1 and 20 AAs and the *neq*_*SL*_ values vary between 1 and 27 SLs. The higher the value of *neq is*, the more the position *is variable*. For a given position, the entropy is maximum when all the symbols AA or SL are equally like. These two exponent of Shannon entropy parameters can differentiate three types of variable position *i*:

- *neq(i)* = 1 characterizes a strictly conserved position. The *C* chains exhibit the same AA (resp. one SL) at position *i*.
- 1 < *neq(i)* < 1.5 characterizes a weakly variable position. The *C* chains exhibit more than one AA (resp. one SL) at position *i*, but one AA (resp. SL) is predominantly observed. This position *i* exhibits certain rare AA (or SL) changes between the *C* chains.
- *neq*(i) *≥* 1.5 characterizes variable positions. Different AAs (resp. SLs) are observed, meaning that position *i* is variable in terms of the sequence (resp. local structure). A position with a *neq ≥ 3*, *indicating that more* than 3 AAs (resp. three SLs) are equivalently observed, corresponds to “highly variable” sequence (resp. structurally highly variable) position, and *with a neq ≥ 5 corresponds to a* “strongly variable” sequence (resp. structurally strongly variable) position.

**Fig 3:**
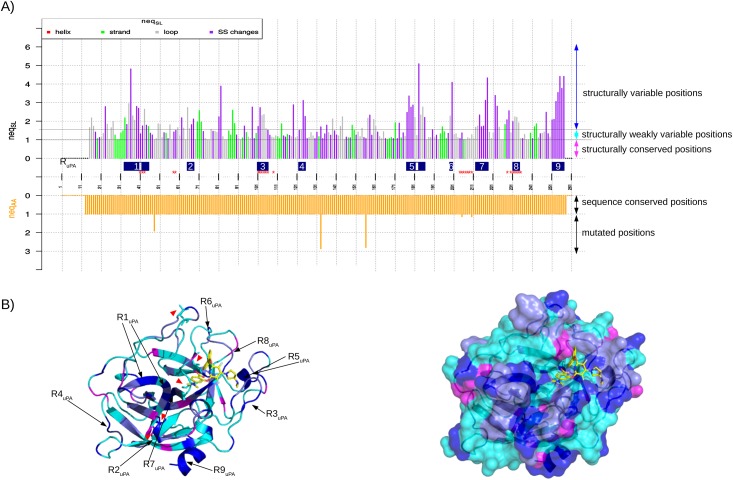
Identification and localization of uPA variable regions. (A) *neq*_*AA*_ (bottom graph) and *neq*_*SL*_ (top graph) values for the 261 positions of the MSA computed using the uPA set (SA-conf step 4 output named Neq_graph.pdf). Bars presenting *neq*_*SL*_ values are colored according to their secondary structure status: the MSA positions in which all chains have an α-helix conformation in red, a β-strand conformation in green, a loop conformation in gray, and where secondary structure changes occur in purple. In this figure, we added blue rectangles to localize the 9 variable regions highlighted during our analysis of the uPA set and defined as regions corresponding to variable positions with at least (i) four (*l = 4*) successive variable positions or (ii) one highest variable position (*neq*_*SL*_ *> 4*): R1_uPA_ (positions 33 − 40 + 42 − 45), R2_uPA_ (positions 65–68), R3_uPA_ (positions 101–106), R4_uPA_ (positions 123–124), R5_uPA_ (positions 177 − 181 + 183 − 186), R6_uPA_ (positions 199–200), R7_uPA_ (positions 212–218), R8_uPA_ (positions 231–234), and R9_uPA_ (positions 251–257). We also added red stars “*” to localize the residues involved in the binding site detected by the PockDrug webserver [46]. (B) Graphical representation of the human uPA domain complexed with a chemical inhibitor (PDB ID: 3IG6). The protein is displayed as a cartoon (left figure) and as a surface (right figure) and is colored according to the structural variability of the positions: structurally conserved positions are colored in magenta, weakly structurally variable positions are colored in cyan, structurally variable positions where SL changes do not imply secondary structure changes are colored in “navy blue”, and structurally variable positions where SL changes imply secondary structure changes are colored in dark blue. Residues located at mutated positions are displayed as sticks. Ligand 438 (HETATM code) is displayed in stick form with its Cα atoms colored in yellow. These figures were generated using PyMOL [61] and the script_pymol.pml generated during the step 5 output. A red triangle was added to easily locate the mutated positions. The presented protein corresponds to the first protein ID in the MSA file. Structurally variable regions are located at the protein surface, except R7_uPA_. R1_uPA_ and R7_uPA_ are within the beta-strand, R2_uPA_, R5_uPA_ and R9_uPA_ include α-helices and R3_uPA_, R4_uPA_, R5_uPA_, R6_uPA_ and R8_uPA_ are within long and solvent-accessible loops.

Values of *neq*_*AA*_ and *neq*_*SL*_ are computed for each position *where* fewer than 50% of the *C* chains have missing residues. The representation of these two parameters for each position (*neq*_*AA*_ and *neq*_*SL*_ *graphics*) results in a joint visualization of the AA and SL variability (Fig 3A). The sequence and structural variability of each position are also indicated below the MSA and MSLA maps, where MSA and MSLA positions are colored according to their *neq*_*AA*_ and *neq*_*SL*_ values. In addition, SA-conf determines the number of secondary structure categories observed at each MSLA position, as determined using the HMM-SA definition [37]. The secondary structure variability is illustrated in the *neq*_*SL*_ graphic, where each MSLA position is colored according to its secondary structure or secondary structure changes (Fig 3A).

#### Step 5—Visualization and localization of variable positions onto a target 3D structure

SA-conf locates the structurally variable positions onto one target structure (the first ID of the MSA output file) based on a script generated using PyMOL software [61]. To do so, this 3D structure is colored according to three structural position types: strictly conserved, weakly variable or variable (Fig 3B). Mutated positions are also highlighted in the stick representation.

### Flexibility analysis in three phases

The analysis of a target flexibility extracted from a set of its MTC using SA-conf operates in three main phases. Phase 1 proposes an overview and description of the 3D conformation of each protein chain. This mining phase can be used on very diverse and large sets to build clean MTC subset(s). Phase 2 results in a simultaneous visualization and comparison of the sequences, secondary structures and local structures associated with all chains of the MTC subset. It provides the quantification of the MTC sequence and structure variability and identification of its variable regions of the MTC set. In phase 3, the user can compare the detected variable regions of one or several MTC set with biological contexts and experimental information, such as partner-interactions, to improve the target flexibility understanding.

#### Phase 1- Mining of 3D conformations

The mining of structure files is performed by combining the outputs of the first three steps of SA-conf. The step 1 provides the description of the submitted structure files. Its output identified the structures that are solved using NMR or X-ray crystallography methods and those corresponding to a monomer (in a free form) or a homo- or hetero-oligomer (in a bound form, complexed with a partner such as protein, nucleic acid, ligand). In addition, the holo forms of the target, i.e., complexed with a ligand, can be distinguished from the apo forms, i.e., not complexed with a ligand, by considering information on the presence of a HETATM (where water molecules are excluded) in each structure file. The combination of this information with MSA and MSLA allows identification of unreliable chains. Protein chain sequences that do not correspond to the target sequence can be identified using the step 1 output table and the MSA. Sequences including mutated positions (deletions, insertions, or substitution), with missing residues or isolated from different organisms can be directly identified using the MSA. Protein chains with particular global or local 3D conformations can be directly detected in the MSLA visualization. This information can be used to prepare a clean MTC set or several MTC subsets of interest to analyze the target flexibility in more detail. Clean MTC subset(s) can be selected, for instance, by detecting and removing the structure chains not corresponding to the target or unreliable ones, retaining the conformation corresponding to one chain in the case of homo-oligomers or selecting MTC corresponding to high-resolution X-ray structures.

#### Phase 2- SA-conf analysis of structural and sequence variability

In phase 2, a deeper and joint sequence and structural variability analysis of the MTC set is performed using the outputs of steps 2 to 5. The MSA and MSLA visualizations allow respectively a global or pairwise comparison of sequence and the local structure chains of MTC. They provide a direct comparison of the sequence and local structural variability of the MTC set. The step 4 output provides a quantification of sequence and structural variability of each position using *neq*_*AA*_ and *neq*_*SL*_ values. Using this information, conserved and variable positions in terms of sequence and structure of the MTC set are easily identified and located. In addition, variable regions of interest defined as *l* successive positions with particular *neq*_*SL*_ values (greater than certain fixed thresholds determined by taking into account the variability of the considered MTC set) can be detected and localized. The visualization of both the sequence and structural variability parameters of each position highlights mutated positions, which are associated or not with structural variations and vice versa. The step 5 output provides a visualization of the structurally conserved and variable positions onto one 3D structure conformation. This allows the localization of structurally variable positions relative to the target surface to determine whether they are accessible to the solvent or buried in the target core.

#### Phase 3- Target flexibility interpretation

In this third phase, the structural and sequence variability information, extracted using one or several MTC subsets obtained under different biological conditions, can offer clues for interpreting the target flexibility. According to the composition of the MTC set, SA-conf can capture different target flexibility, such as intrinsic flexibility and induced-fit effects.

For instance, the joint analysis of sequence and structural variability of a set composed of wild-type and mutant MTC allow for backbone deformation analysis in terms of mutation effects. A position where both AA and SL changes occur together probably corresponds to a mutated position involving a backbone deformation. If one position is variable in SL but not in AA, the observed backbone deformation can be imputed to the intrinsic flexibility of the target, to partner interactions or to experimental condition variations, according to the composition of the MTC set. In contrast, if one position is variable in AA but not in SL, the side-chain change likely has no effect on the backbone conformation of the corresponding residue and its direct neighbors. However, it is possible that this mutation involves an “indirect” backbone deformation via interactions with close residue in the 3D space. It can be suggested by the visualization of the 3D structure (step 5 output).

SA-conf results obtained using a subset of MTC in free form with an identical sequence enable the identification of the structural variation corresponding to the intrinsic flexibility or certain experimental condition variations (pH, space group, etc.). This MTC set can correspond to structures obtained using NMR or molecular modeling techniques.

To identify induced-fit structural changes, structurally variable regions obtained using a MTC set included bound forms can be matched with binding regions extracted by the user. This can aid in the characterization of variable regions involved in protein function and/or interactions with partners. A comparison of SA-conf results obtained using MTC complexed with different protein or nucleic partners might aid in identifying structural deformations involved in protein or nucleic-acid binding. A comparison of SA-conf results obtained on MTC bound to different ligands might help identify structural deformations induced by ligand binding and ligand diversity.

### SA-conf implementation

SA-conf is a freely available combined Python and R programming language. that run on a GNU/Linux system. These programs require Python [62], the Biopython Python package [63] and working installations of the R [64], PyMOL [61], ClustalW [53] and T-coffee [54] programs. The underlying data and SA-conf executable script, with a set of pre-compiled binaries are available at http://www.mti.univ-paris-diderot.fr/recherche/plateformes/logiciels.

Even if the duration of the SA-conf process depends on the number and length of the considered MTC, SA-conf is a notably quick tool that can analyze a large dataset. For example, the run of SA-conf using as input the “uPA set” (105 PDB chain IDs) with a pre-computed MSA on the same computer lasted 3.09 min in Ubuntu on an Intel R Xeon(R) CPU E5-2609 0 @ 2.40 GHz x 8 processors.

## Results and illustrations

SA-conf tool was applied to different MTC subsets associated with three targets uPA, p53 and PR1 (Table 1) with the aims of i) mining the available 3D conformations associated with each target and selecting pertinent MTC subset(s), ii) identifying the variable regions of interest of MTC subsets in terms of the 3D local structures, secondary structures and sequence, and iii) providing insights into flexibility of each target by combining the SA-conf variability results obtained using its different MTC subsets.

### Conformation set mining

To capture the target structural variability without introducing bias into the analysis, it is essential to work on a clean MTC subset. SA-conf is able to mine diverse 3D conformation sets and is used to firstly prepare clean subsets, as illustrated below on uPA and p53 targets.

To build an uPA MTC subset, we first ran SA-conf on a list of 107 PDB IDs corresponding to UniProt ID P00749 (human uPA). The structure overview provided by SA-conf highlighted the heterogeneity of the structure set. It includes a mixture of 54% monomers and 45% oligomers with certain heteromer complexes, and most of the structures are complexed with a ligand. The SA-conf MSA showed that some of the 187 chains are mutant forms and only 105 chains matched with the uPA catalytic domain (S3 Fig). A clean MTC set, “uPA set” was built by manually selected these 105 uPA chains. It includes chains in apo and holo forms, wild-type and mutant sequences (Table 1).

To build a clean heterogeneous MTC p53 DBD subset, we first ran SA-conf on a heterogeneous list of 78 PDB IDs associated with UniProt ID P04637 (70 human cellular tumor antigen p53) and UniProt ID P02340 (8 mouse cellular tumor antigen p53). The SA-conf MSA highlights several chains that do not match with the p53 DBD sequence (S4 Fig). We built the “P53 set” by retaining only one chain by PDB files matching with p53 DBD sequence. This set correspond to a heterogeneous subset composed of 76 X-ray and two NMR structures (PDB IDs: 2FEJ and 2MEJ), in bound (complexed with different partners such as p53 DBD, another protein, DNA or small molecule) or free form, corresponding to wild-type or mutant forms and human or mouse p53 DBD chains (Table 1).

### Detection of variable regions

We explored the flexibility of the three targets using SA-conf by locating and quantifying the sequence and structural variability of each considering MTC set. The MSA maps obtained using the uPA and P53 sets showed that some positions are misaligned (data not shown). Thus, we re-ran SA-conf on the uPA and P53 sets using a manually corrected MSA. Using SA-conf output, we observed that the three targets exhibit few sequence variability with an average *neq*_*AA*_ fewer than 1.1 (Table 2). The associated MSA maps (Figs 2A and S5A) highlight and localize the chains of each subset containing mutations, unresolved or modified residues. Thus, using MSA map, it is easily to identify the target chains associated with particular sequences, such as the last eight p53 mouse sequences (S5A Fig).

**Fig 4:**
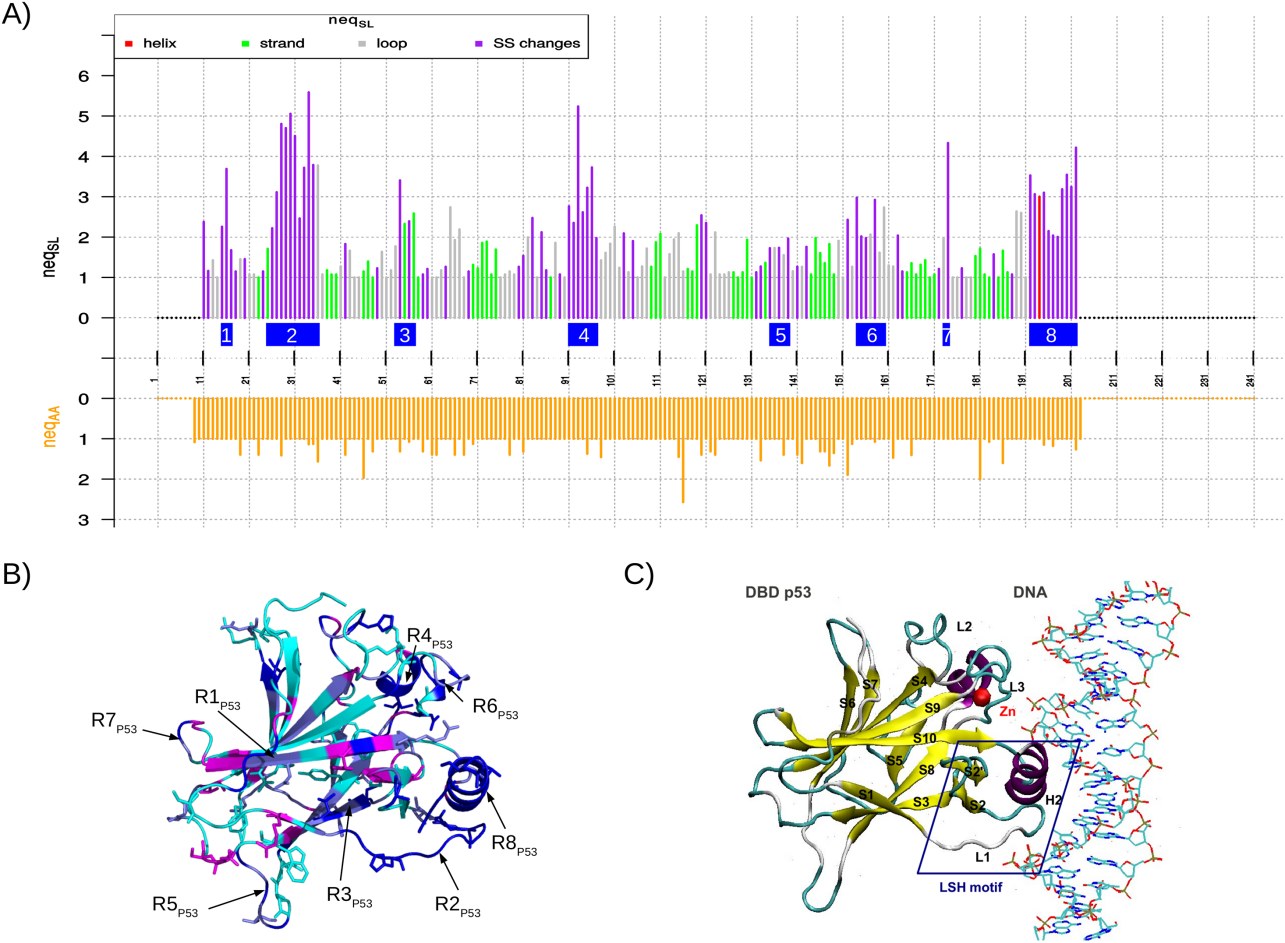
Identification and localization of the p53 DBD domain using the P53 MTC set. (A) *neq*_*AA*_ (bottom graph) and *neq*_*SL*_ (top graph) values for the positions of the MSA computed using the P53 set (SA-conf step 4 output named Neq_graph.pdf). Bars presenting *neq*_*SL*_ values are colored according to their secondary structure status: the MSA positions in which all chains have an α-helix conformation in red, a β-strand conformation in green, a loop conformation in gray, and where secondary structure changes occur in purple. In this figure, we added blue rectangles to localize the eight variable regions highlighted during our analysis of the P53 set and defined as regions corresponding to variable positions with at least (i) five (*l = 5*) successive structurally variable positions or (ii) one highly variable position (*neq*_*SL*_ *≥ 3*). They are labeled from R1_P53_ to R8_P53_ and correspond to respectively positions 15–17, 25–36, 53–57, 91–97, 135–139, 154–160, 173–174 and 192–202. We also added red stars “*” to localize the residues involved in the binding site detected by the PockDrug webserver [46]. (B) Graphical representation of the human p53 DBD domain complexed with the Large T antigen (colored in gray). (PDB ID: 2H1L). The protein is displayed as a cartoon (left figure) and as a surface (right figure) and is colored according to the structural variability of the positions: structurally conserved positions are colored in green, weakly structurally variable positions are colored in cyan, structurally variable positions where SL changes do not imply secondary structure changes are colored in “navy blue”, and structurally variable positions where SL changes imply secondary structure changes are colored in dark blue. Residues located at mutated positions are displayed as sticks. Ligand 438 (HETATM code) is displayed in stick form with its Cα atoms colored yellow. These figures were generated using PyMOL [61] and the script_pymol.pml generated during the step 5 output. A red triangle was added to easily locate the mutated positions. The presented p53 DBD corresponds to the first protein ID in the MSA file. Region R1_P53_ is located in the N-terminal region, regions R2_P53_, R4_P53_, R5_P53_, R6_P53_, R7_P53_ are located in loops, R3_P53_ is located in a β-strand, and R8_P53_ is located in an α-helix. (C) Structural description of the human p53 DBD domain complexed to DNA (PDB ID: 1TSR). The protein is displayed as a cartoon and is colored according to its secondary structures: α-helices in purple, β-strands (named S1 to S10) in yellow, turns in cyan and loops in gray. Loops L2 and L3 are coordinated with a structural zinc ion (sphere in red). The LSH motif, shown in the blue frame, is composed of helix H2, loop L1 and beta hairpin S2-S2'. This motif and the L3 loop are directly involved in DNA interactions.

SA-conf provides a quantification of the structural variability for each aligned position using entropy parameter (*neq*_*SL*_) that allowed us to locate structural variable positions in each MTC subset (Figs 3A, 4A and S7). SA-conf results show that each MTC set (except the two p53 homology model sets) exhibit relatively large global structural variability with an average *neq*_*SL*_ larger than 1.5 and more than 20% of structural variable positions (Table 2). The MSLA maps, computed on the heterogeneous uPA, PR1 and P53 sets, show rather similar local conformations (in terms of SLs) for a particular MTC set but highlight some particular conformations (Figs 2B, S5 and S6), such as those exhibiting by the NMR models of the PR1 and P53 sets (S5 and S6 Figs). By considering the successive structural variable positions, we identified from seven to nine variable regions in each MTC set (Table 2, Figs 3A, 4A and S7). For each subset, we defined most variable regions according to the variability of the studied dataset. For instance, nine most structurally variable regions were detected from uPA set (Fig 3A) and defined as regions with at least four *(l = 4)* successive variable positions or at least two *(l = 2) with* one highest variable position (*neq*_*SL*_ > 4). Using the SA-conf output that locates structural variables position onto one uPA structure, we observed that four of them are short loop fragments, included within long and solvent-accessible loops (Fig 3B and 3C). Thus, these long loops are composed of a succession of weakly and highly variable regions in agreement with Regad et al. (2010) who showed that the long loops are not random coils [38].

SA-conf also allows the detection of structural variability in terms of secondary structure changes. From the different MTC subsets (except the homology model sets) about 40% of the structurally variable positions exhibit to the same secondary structure (loops or β-strands) in all structures (S1 Table). This highlights that the detailed SL description of local 3D structures enables the detection of backbone deformation, not captured by the classical secondary structure information [35].

### Structural variability interpretation

SA-conf allowed us to detect structural variable positions and regions in the different subsets for the three targets. In the following, we illustrated how the analysis of SA-conf results obtained on adequate MTC sets and crossing these results with known information about target allows capturing and distinguishing the different sources of structural variability.

#### Extracting structural variability and mutation relationship

Mining a MTC set composed of both wild-type and mutant conformations provides a simultaneous analysis of sequence (mutated residues) and structural variable positions. Thus, by comparing these positions using the join representation of *neq*_*AA*_ and *neq*_*SL*_, it is possible to identify if mutations induce direct backbone deformations. For instance the five mutated positions detected in the uPA set are structurally conserved, i.e. exhibiting a small *neq*_*SL*_ value, and not involved in direct backbone deformation (Fig 3A). However long-range effects of a mutation could be identified by the visualization of the 3D structure provided by SA-conf. For instance, the visualization of uPA 3D structure (Fig 3B) shows that one mutated position is close in space to one variable region’s position (less than 3 Å). This suggests that the structural variability of this region could be induced by the mutated position.

Using the heterogeneous P53 set, among 86% of structurally variable positions only a small portion of structural variability can be directly linked with mutations (76% of conserved positions in sequence). We note that two of known oncogenic mutations, occurring at aligned positions 158 and 195 (G245S and R282) are located in variable regions R6_P53_ and R8_P53_ (Fig 4). This suggests they might have a direct impact on the backbone deformation, as described by Calhoun and Daggett (2011) [65]. Other oncogenic mutations may have an indirect impact on the structure stability via long-range interactions, in agreement with two classes of oncogenic mutations in the p53 DBD: (i) those that directly affect the p53 DBD binding to DNA and (ii) those that destabilize the protein conformation and have a more indirect effect [47,66]. The link between the sequence evolution and the structural variability of a target can also be studied by SA-conf mining of a MTC set of homologous proteins. For example, the joint analysis of the P53 set showed that the mouse chains are clearly different in sequence from human chains (S5A Fig). However, at the global or local structure level, this difference is no longer perceptible (S5B Fig). The weak backbone deformations induced by these mutations were in accord with the permissive mutations between two mammalian proteins that weakly affect the backbone structure properties [67].

#### Extracting structural variability caused by induced-fit effects (partner-binding)

In this section, we illustrate that mining an appropriate subset of MTC using SA-conf allows detecting structural backbone deformations resulting from partner binding. This requires that the treated MTC set contains both free and bound (with different partners) forms of the target, such as the heterogeneous uPA, P53 and PR1 subsets. The analysis of SA-conf results allowed the detection of structural variable regions in each submitted subset. Then, to identify backbone deformation induced by partner binding in each subset, we compared the detected variable regions with partner-binding regions described in the literature or that we previously detected (see Materials & methods).

In the uPA set, half (48%) of the pocket residues are structurally variable and located within or close in space to three of nine detected most variable regions (Fig 3A, S2 Table). This suggests that the binding of diverse ligands leads to the backbone deformation in some pocket residues. On the opposite, some pocket residues involved in ligand hydrophobic interactions (S2 Table) are structurally conserved between the apo and holo conformations suggesting that they could be “key” residues for binding (Fig 3A). These results confirm that the binding pocket of uPA is composed of both rigid residues important for the biological function of the target and flexible residues involved in the adaptation of the pockets to different ligands.

Using the PR1 set, six of the eight most structurally variable regions detected contain residues of the dimerization interface. The one located within the flap region contains pocket residues (S7 Fig). Two other most variable regions match with the flap elbow and the end of the flap regions, known to be involved in the closeness of the binding site upon ligand binding. The last most variable region fits with the cantilever and α-helix regions. Thus, this suggests that the binding of diverse ligands in PR1 induces backbone deformation in some pocket residues but also has an effect on the dimerization interface.

Using the P53 set, five of the eight most structurally variable regions detected match with the well-known partner-binding sites, including DNA, protein, and small-molecule-binding sites (Fig 4). As the P53 set is composed of bound and free forms, we conclude that part of the structural variability of some detected p53 variable regions is linked to different partner binding. For the three targets, some structurally variable regions detected by SA-conf match with functional regions. Thus, the identification of structural variable regions by mining a MTC set using SA-conf can help to suggest functional regions and can help to determine the target function.

#### Extracting the intrinsic structural variability

To detect the PR1 and p53 intrinsic flexibility, we applied SA-conf to homogeneous subsets (P53-NMR, P53-HM_QM_, P53HM_HM_, PR1-NMR), which exhibit no sequence changes, in the same form (complexed or not with a partner), and determined with identical resolution method.

**Fig 5:**
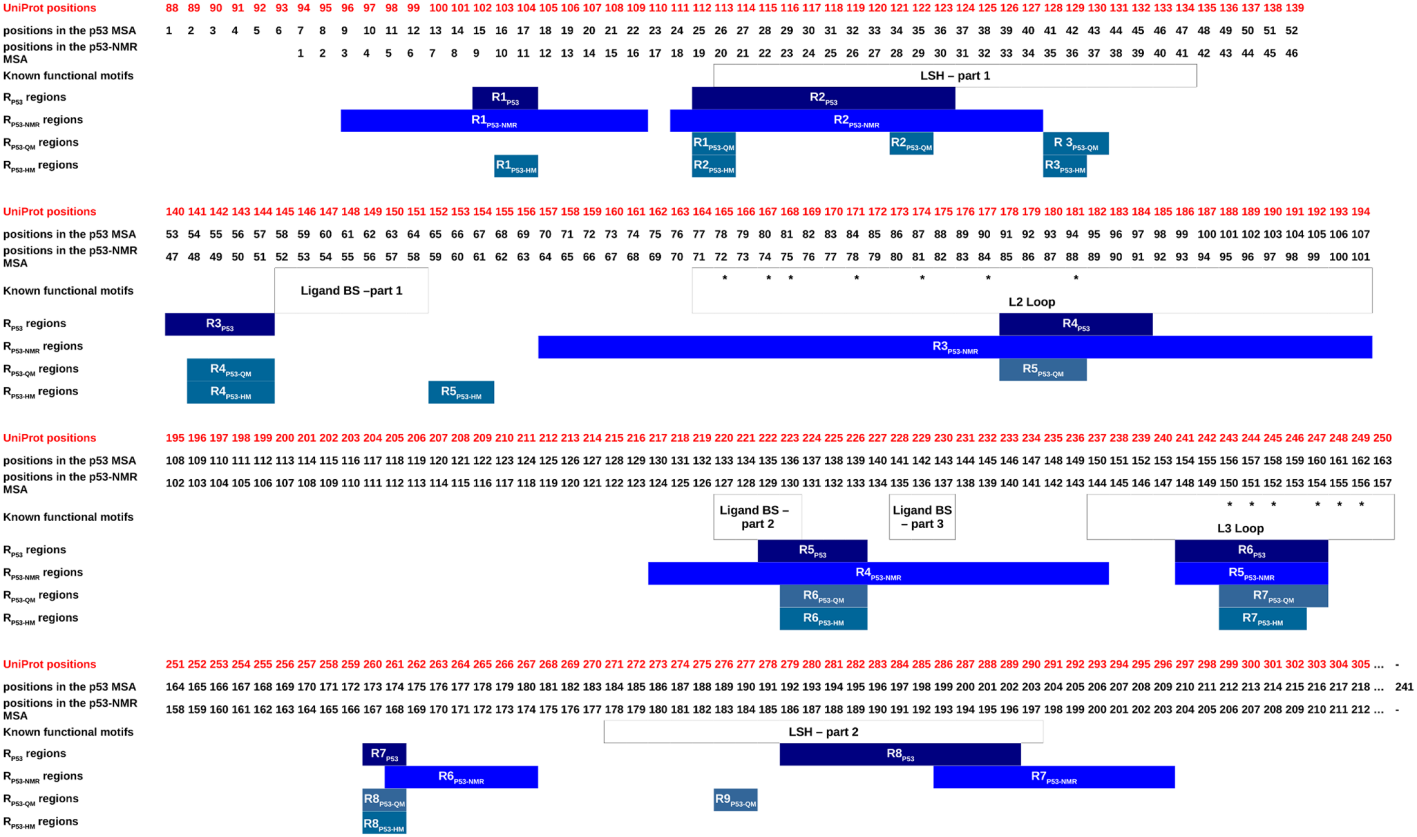
Correspondence between known functional motifs of the p53 DBD domain and the variable regions detected using different p53 MTC sets: The P53, P53-NMR, P53-HM_HM_, and P53-HM_QM_ sets. L1, L2, and L3 loops are involved in direct DNA interactions. The L2 and L3 loops are also involved in the interaction with the 53BP1 protein [47]. Residues involved in the interface between p53 DBD and 53BP1 proteins were estimated using 2P2I Inspector server [68] with the 1GZH PDB ID as input. They are identified by “*”. Ligand-binding site was estimated using PockDrug server [46] with the 4AGM PDB ID as input. The seven structural variable regions extracted from the P53-NMR set are named R1_P53-NMR_—R7_P53-NMR_. They correspond to regions with at least (i) seven (*l ≥ 7*) successive variable positions or (ii) one strongly variable position (*neq*_*SL*_ *≥ 5*). The eight and nine structural variable regions extracted from the P53-HM_HM_ and P53-HM_QM_ sets are respectively named R1_P53-HM_ − R8_P53-HM_ and R1_P53-QM_ − R9_P53-QM_. They correspond to regions with at least two (l ≥ 2) successive variable positions.

SA-conf detects a large intrinsic structural variability using the P53-NMR set (average *neq*_*SL*_ = 2.69, Table 2) with seven long and strongly variable regions (Figs 5 and S8). One of the three most variable regions is inside the L2-loop (S8 Fig). This particularly large flexibility of the L2 loop is expected owing to its long length of 30 residues. Five of these variable regions overlap with partner-interaction regions (Figs 5 and S9), such as the DNA-binding region [47] and regions close to the ligand-binding site [50]. This is in agreement with the intrinsic flexibility of the interaction regions detected in the literature and the well-characterized p53 DBD flexible regions observed using molecular dynamics simulations [65,67]. For instance, Calhoun and Daggett (2011) compared cancer-associated mutant with the wild-type p53 DBD using molecular dynamics experiments [65]. These authors highlighted large fluctuations and deviations of the L1 loop and of the loop including binding site portions (UniProt residues 223–230), corresponding both to one variable region. All these results suggest the existence of intrinsic flexibility to facilitate the interactions.

We also extracted p53 intrinsic flexibility by analyzing SA-conf results obtained on the two homology modeling sets P53-HM_HM_ and P53-HM_QM_ (Table 1). These two sets exhibit a weak structural variability associated with short structurally variable regions matching roughly with intrinsic flexibility detected using P53-NMR (Fig 5, Table 2). This is partly explained by spatial restraints and structural proximity constraints associated to Modeller software. We note that detected variable regions do not contain mutated positions reverted to wild-type residues, respectively four and six in P53-HM_QM_ and P53-HM_HM_, but their shortness could result from the expected mutations stabilization effect on the domain [69,70].

**Fig 6:**
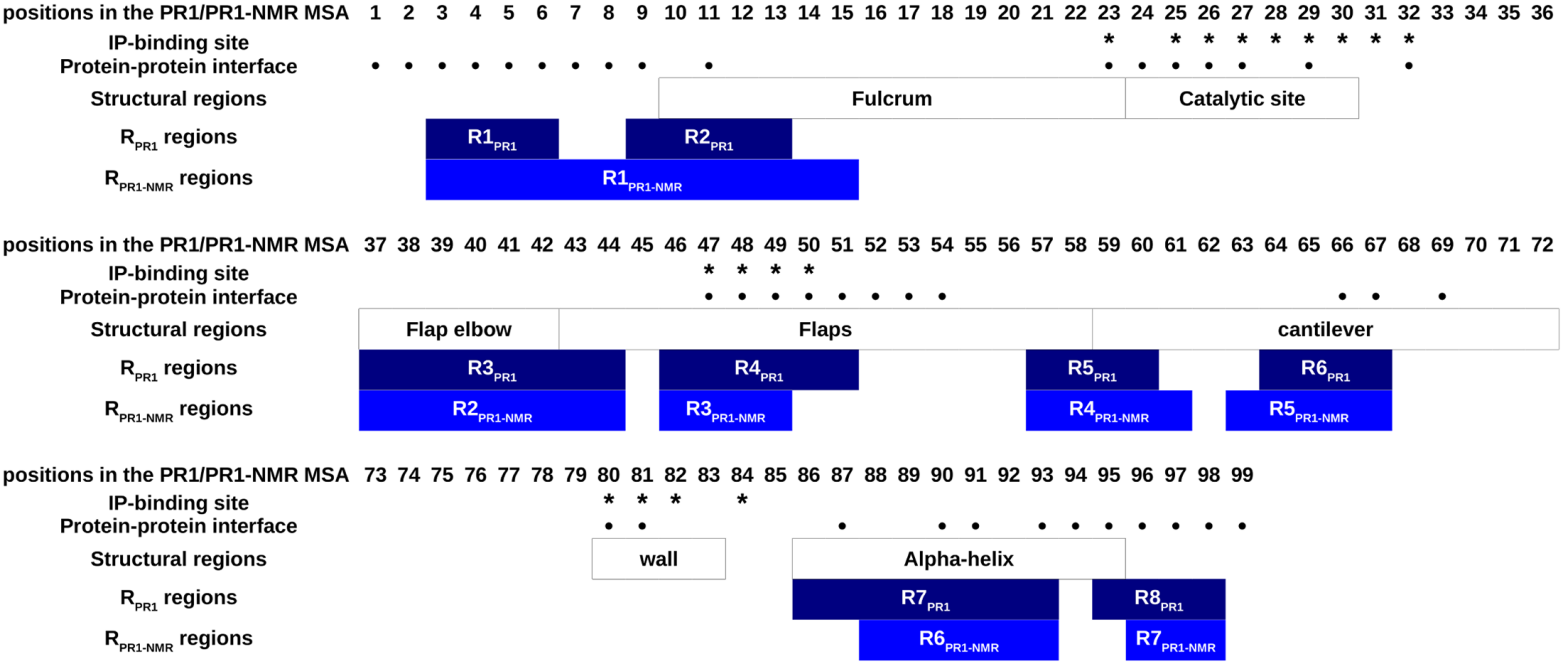
Correspondence between known important PR1 regions and detected variable regions using PR1 and PR1-NMR sets. Residues involved in the dimerization interface of PR1, estimated using 2P2I Inspector server [68] with the 1HXB PDB ID as input, are identified by “●”. Ligand-binding site was estimated using PockDrug server [46] with the 1HXB PDB ID as input, are identified using “*”. Structural regions correspond to regions defined by Sadiq et al. (2010) [72]. The eight and seven structural variable regions extracted from the PR1 and PR1-NMR sets are respectively named R1_PR1_-R8_PR1_ and R1_PR1-NMR_-R7_PR1-NMR_. They correspond to regions with at least (i) four (*l ≥ 4*) successive variable positions or (ii) one strongly variable position (*neq*_*SL*_ *≥ 3*).

Using the PR1-NMR set, SA-conf highlights seven most variable regions, particularly variable when corresponding to the well-known flexible flap elbows and flap regions (Figs 6 and S8). This is in agreement with the strong flexibility of these regions obtained using molecular dynamics simulations by [71]. In addition, we highlighted two strictly conserved regions, matching with the wall region and the catalytic site in accord with the biological function of the catalytic site that requires a particular conformation.

#### Extracting target flexibility insight by crossing MTC subset variability results

**Fig 7:**
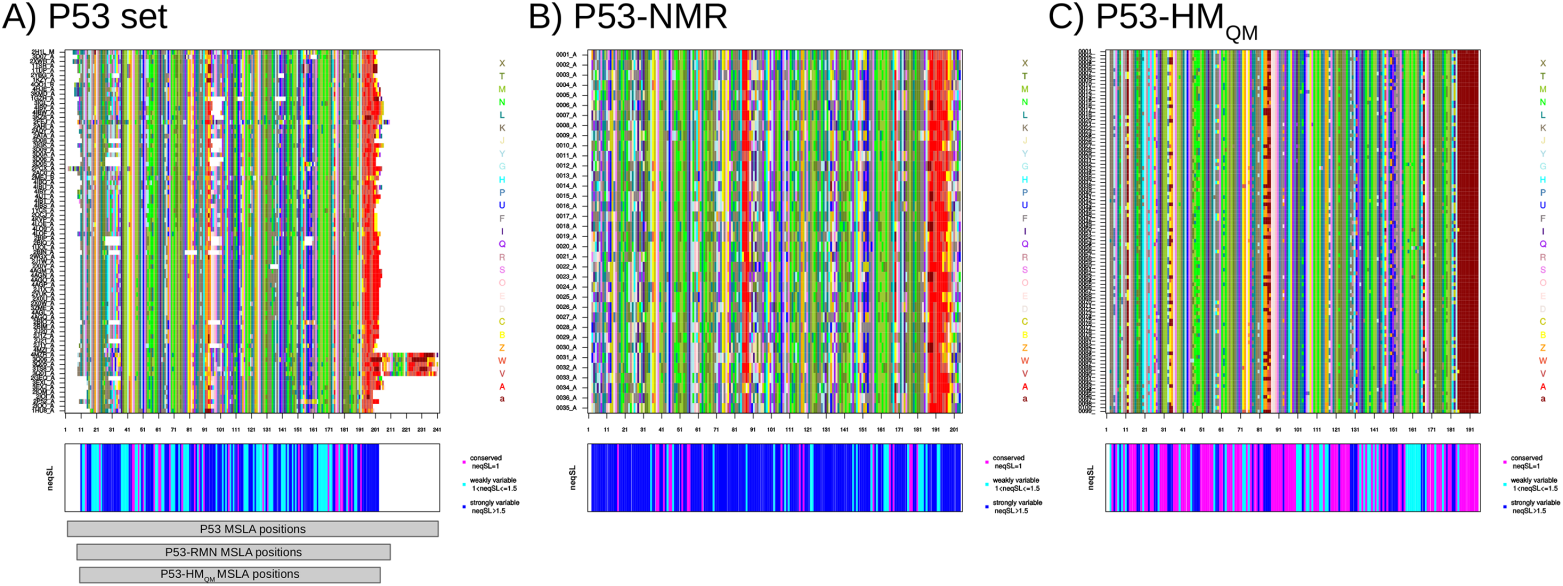
Visualization of the structural variability of three subsets associated with the p53 DBD: For the P53 (A), P53-NMR (B), and P53-HM_QM_ (C) sets. The upper figures correspond to the MSLA maps computed for each subset. The aligned SL sequences are shown in rows and the aligned positions (in columns) are colored according to the 27 SLs. The colors of the SLs indicate the associated secondary structure: [a, A, V, W]-SLs in red are primarily found in the α-helix, [L, M, N, T, X]-SLs in green are primarily found in the β-strand, other SLs correspond to loop. The bottom figures present structural conservation/variability of each subset position in terms of *neq*_*SL*_. Positions are colored according to their *neq*_*SL*_ values. Structurally conserved positions, i.e., positions exhibiting a *neq*_*SL*_ value of 1, are colored in magenta. Structurally weakly variable positions, i.e., positions exhibiting a *neq*_*SL*_ value included from 1 to 1.5, are colored in cyan. Structurally strongly variable positions, i.e., positions exhibiting a *neq*_*SL*_ value larger than 1.5, are colored in blue. The alignment of positions of the three sets is presented below graphs of (A).

Previously, we observed that the analysis of an adequate MTC set allows us to detect structural variability induced by different reasons. By combining the variability information extracted from different MTC subsets, it is possible to differentiate the different flexibility types. We compared SA-conf results obtained using three p53 subsets (p53, p53_NMR_, and P53-HM_QM_) to investigate the structural variability induced by two different experimental (X-ray and NMR) or one theoretical resolution methods (homology models). First, the average *neq*_*SL*_ value of each set showed that the P53-HM_QM_ set exhibits less structural variability than relative to X-ray structures and NMR sets. In addition, the three obtained MSLA shows different local structural variability related to each resolution method used (Fig 7). Indeed, less structural variability is observed from p53 chains obtained by homology modeling relative to X-ray structures and NMR methods (Table 2). In more detail, some particular local conformations are sampled by different resolution methods used (Fig 7). For instance, the p53 homology models tend to result in very regular C-terminal α-helix conformations and the p53_NMR_ set have structures with particular conformations at some positions relative to other structure set. These particular local conformations can be resulted from the resolution method. This highlights that conformational variability depends on the technique used to generate the structures. Then the comparison of SA-conf results can be pursued to know if the structural variable positions in each set are located in the same regions and if they exhibit the same structural variability magnitude. The analysis of the overlap between the most structural variable regions of each set (Fig 5) shows that most of structurally variable regions of the three sets overlap indicating that the different techniques used to resolve protein structures yield different structural variability that finally converge to identify similar variable regions. However, the p53_NMR_ set have regions more variable in length and magnitude than the P53 set. This result can be partly explained by the greater number of constraint conformations in the heterogeneous P53 set due to the crystallographic technique. These local and global differences detected by SA-conf between p53 X-ray and NMR conformations, have also been reported by Lukman and collaborators on a large p53 DBD conformer dataset [67] or on a large diverse set [73]. In addition, in all p53 MTC subsets except in the P53-NMR set, one short variable region (close to the ligand-binding site) is observed. This suggests that the variability of this region is most probably explained by the deformation involved by ligand interactions, not observed using the P53-NMR set.

All seven identified P53-NMR variable regions match with a portion of the heterogeneous P53 variable regions and five are located within the interaction regions (Fig 5). The comparison of the P53 and P53-NMR set variability highlights intrinsic loop flexibility but certain stronger conformation changes correspond to variability induced by DNA binding or different ligand binding using the P53 set (Fig 5). Interestingly, in all p53 MTC subsets except in the P53-NMR set, one short variable region (close to the ligand-binding site) is observed. This suggests that the variability of this region is most probably explained by the deformation involved by ligand interactions, not observed using the P53-NMR set. Deeper SA-conf analyses could be pursued by building other p53 DBD subsets, such as those composed of only bound structures or only oncogenic mutations, and by comparing them with a free wild-type DBD p53 set (X-ray or NMR). Concerning PR1 target, the comparison of the MSLA obtained using the PR1 set showed that NMR models exhibit particular local conformations (S6 Fig). As for p53 target, seven identified PR1-NMR variable regions match with a portion of the PR1 variable regions (Fig 6).

We note despite some local structural differences detected for MTC resolved using different resolution methods, SA-conf globally detects similar variable regions for the different MTC subsets associated with P53 or with PR1 target (Figs 5 and 6). The overlap of the detected structural variable regions despite the MTC subsets for p53 or PR1 targets is in agreement with the similar variability detected in the ubiquitin target using various X-ray structures, NMR models and molecular dynamics simulations [74,75]. For the two PR1 and p53 targets, our results show that intrinsic flexible regions match with flexible regions explained by partner-binding effects, suggesting the existence of intrinsic flexibility to facilitate the interactions.

## Discussion and conclusion

We demonstrated the importance of exploring the variability of several 3D structures associated with a target to provide flexibility insight. This was performed using three well-characterized targets chosen for their large number of available structures under variable conditions (e.g. experimental resolution, partner binding, mutations). For this purpose, we developed a new SA-based tool, which facilitates the analysis and comparison of large and diverse MTC sets and exploration of available structural variability landscape associated with a given target. SA-conf quantifies the variability of any MTC set by taking into account the three levels of protein descriptors, i.e. sequence, secondary structures and 3D local structures.

SA-conf tool is put in context with some other programs and software in the Introduction. More specifically, and as described in [19], the existing visualization programs such as PyMOL [61] can provide sequence or structure alignment but are not suited for the automation and comparison of a large MTC set. Molecular mechanics programs such as GROMACS [76] and CHARMM [77] analysis can provide structural quantification for structural ensembles most often associated with a molecular dynamics simulation but are expensive with respect to time and can be performed only on a restricted number of target forms. Other programs or web servers dedicated to the computation of multiple structure alignments such as MUSTANG-MR [13] are able to analyze two or more structures with different sequences but with a high computational cost. Based on a SA simplification, recent multiple structure alignment methods, such as MulPBA [14], reduce the computational cost but can have still some difficulties in obtaining global convergence and providing a good multiple structural alignment using a large and diverse dataset. Additionally, these approaches do not provide structural variability quantification.

Considering tools dedicated to the analysis and quantification of the structural variability, we found three direct competitors able to obtain automatic, precise, and detailed structural statistics of MTC sets: CoDNaS [16,18], GSA-tool [23], PSSweb [19,20]. A detailed comparison of the technical features of these four tools dedicated to the structural variability analysis is presented in Table 3.

**Table 3:** Criteria for the comparison of SA-conf and other software dedicated to the structural variability analysis and quantification of a set of MTCs.

	CoDNaS [16,18]	GSA-tool [23]	PSSweb [19,20]	SA-conf	
Dataset	Difference sequences	No (sequences must have more than 95% sequence identity with the target)	No	Yes	Yes
	Multimer	No		Yes	Yes
	Heterogeneous dataset (MTCs generated using different methods)	Yes: MTCs extracted from the PDB	No: Models generated using molecular dynamics	Yes: All MTC structures	Yes: All MTC structures
Choice of a reference		No	No	Yes	No
Approach to comparing MTCs	Pairwise comparison	Yes	No	No	Yes
	Comparison of all MTCs	No	Yes	Yes	Yes
Criteria to quantify structural variability by position	Computation of the average variability			Yes	No
	Computation of the number of possible local structures			No	Yes
Protein description used to compare MTCs	AA	No	No	Yes (no output)	Yes
	SS	No	No	No	Yes
	3D	Yes	Yes	Yes	Yes
	Bfactor	Yes	No	Yes	No

CoDNaS [16,18] is a database of protein conformational diversity that analyses the structural diversity of a protein’s native state available in the PDB. GSATools explores the conformational space of target models determined using molecular dynamics simulations based on a SA [23]. Consequently, it focuses on the analysis and quantification of the intrinsic structural variability of an MTC with identical sequence and obtained under the same conditions. Pandini et al, 2016 [33] recently concluded on the advantages of combining molecular dynamics simulation and knowledge-based on a SA to increase the exploration of the conformational space of proteins because observing a conformational change of a protein is difficult and often requires lengthy computation time. CoDNaS extracts from the PDB the conformers of a target corresponding to chains that share more than 95% sequence identity with the target. One advantage of SA-conf relative to these two approaches is that it is capable of analysing very diverse sets associated with a target composed of structures with no constraints on the similarity of sequences, extracted from different sources and complexed with different partners. In addition, SA-conf can be used on NMR and crystallographic structures, on theoretical models obtained using modelling techniques or on MTC sets obtained by molecular dynamics simulations or using a generator of native target ensembles in statistical thermodynamic terms such as COREX/BEST server [78].

PSSweb [19,20] is a webserver dedicated to an automatic and detailed statistical analysis of a large set of MTCs with various sequences and 3D conformations. PSSweb is based on the MTC superposition onto a pre-defined reference and calculates the standard deviation of the backbone or side-chain atom coordinates (*rmsf*) or dihedral angles for each aligned position. To determine the structural variability of a MTC set, PSSweb determines also the average B-factor value for each position. This parameter that reflects the degree of thermal motion and static disorder of an atom in a protein crystal structure [79] is also used in CODNAS program. To compare PSSweb and SA-conf results, we computed the Pearson coefficient correlation values between the average PSSweb B-factor per residue and SA-conf *neq*_*SL*_ values of each position in the three sets containing X-ray structures: uPA, P53 and PR1 sets. The obtained Pearson coefficients are relatively low and vary between 0.43, 0.48 and 0.28, respectively. This is in accord with Dong et al. results (2016) that showed that the correlation between the conformation entropy computed using different SA and the protein flexibility quantified using B-factor values is dependent on the studied proteins [61]. Moreover, this B-factor parameter is estimated only on X-ray structures and can be difficult to compare from different structures as influenced by many factors such as the overall resolution of the structure and, importantly, the particular refinement procedures [80,81]. To pursue the comparison between PSSweb and SA-conf results, we computed the Pearson coefficient correlation values between the *rmsf* and *neq*_*SL*_ values of each position in the seven uPA, p53 and PR1 subsets (S3 Table). The well-known metric *rmsf* calculated by PSSweb exhibits variable correlation coefficients ranked from 0.01 to 0.77 on the different subsets. These results demonstrate that *rmsf* and B-factor parameters quantify complementary information from the exponent of Shannon entropy. The SA-conf exponent of Shannon entropy presents the interest to measure both the quantity of average information of a position and its uncertainty in terms of observed local conformations. Hence, this criterion is effective to detect highly variable positions as an *rmsf* metric but also positions that exhibit little variability. In this way, the exponent of Shannon entropy is a particularly adapted criterion for mining unbalanced or representativeness protein datasets. It can detect structural variability information due to a few particular observed conformations of a target, and the associated MSLA map then allows a direct identification of the particular conformations.

SA-conf tool is based both on a MSA computed using all sequences of MTC and on MSLA deduced from this MSA using HMM-SA simplification. The comparison of the corresponding SL sequences avoids the 3D superposition of MTC structures, which makes SA-conf robust to particular conformations. However, as in PSSweb, the structural variability quantification in SA-conf is based on an MSA computation. Thus, the structural variability quantification quality depends on the quality of the MSA, which must be carefully performed and manually corrected if necessary. To illustrate the importance of the high-quality MSA, we ran SA-conf on the P53 using an MSA that we randomly moved a gap (amongst the 3484 gaps of the set: on average a p53 DBD aligned sequence has 45 (± 11) gaps). A gap put on the beginning or the end of the MSA has no effect on the SA-conf results. However, a gap put within the MSA between two AA modifies the MSA and thus the MSLA, that results in some sequence and structurally conserved positions become variable. For instance in two simulations where a gap were shifted between two AA, 60% of conserved AA positions become mutated positions and more than 65% of structurally conserved positions become weakly variable. These results show that misalignment in the MSA lead to bias in the SA-conf results. This highlights the importance to check the quality of the computed or submitted MSA before the analysis of SA-conf results.

Finally, it is demonstrated that SA-conf is efficient to detect putative structural deformation induced by a partner binding when it was applied to heterogeneous MTC set and intrinsic variability when it was applied to homogeneous MTC set.

The illustrations confirmed that different techniques used to resolve protein structures yield different structural variability that finally converge to identify similar variable regions. Despite the structure resolution methods used, the coherency of the detected structural variable regions confirms that variability information can be crossed to *provide insight and interpretation of different sources of target flexibility*. *Moreover*, we confirmed the interest in studying protein diversity and redundancy to identify structurally variable regions located in the partner-binding site regions. Our analyses of the structural variability of p53 and PR1 targets emphasize that the structural backbone deformation of regions involved in interaction results from both induced-fit effects and intrinsic variability. This suggests that intrinsic flexibility is important and necessary for some partner binding to occur. Putative induced structural deformation due to the partner binding can be detected using heterogeneous MTC set while intrinsic variability can be detected using homogeneous MTC set. The comparison of SA-conf variability results obtained using different MTC subsets associated with the same target can offer interesting clues to better understand its plasticity and function. This work demonstrates the interest to study different structures of the same target, such as NMR, crystallographic structures and homology models. However, the MTC sets associated with a target and available in the PDB can severely under-sample the whole target space and can provide uncompleted and erroneous structural variability information. In the case of P53, SA-conf tool applied on combined Xray, NMR and homology models data highlights similar results to those obtained on MTC sets obtained by molecular dynamics simulations [65,67]. The advantage of SA-conf is that is can be apply to structure sets available in the PDB (crystallographics and NMR structures), but also to theoretical models such homology models and to MTC obtained using dynamics simulations to analyze a large conformational space of the target. Finally, this work underlines that structural variability analysis is an important source of structural knowledge of the protein universe.

## Supporting information

S1 Fig Preparation of the MTC set to analyze the structural variability of the human p53 DBD using NMR models extracted from one PDB file. 3D coordinates of each NMR model are extracted from the PDB files and stored in a text file in PDB format, which the name correspond to an artificial PDB ID (four characters + “.pdb”). The artificial name of each created PDB file are stored in a text file. This text file and all created PDB files will be the input of the SA-conf software, as illustrated for the 2FEJ PDB file.
(TIFF)

S2 Fig Encoding of the 3IG6_A chain structure using HMM-SA. This figure presents the simplification of two protein chains (3IG6_B and 3KGP_A) into SL sequences using the structural alphabet HMM-SA. Each protein structure is displayed as a cartoon and colored according to the 27 SLs. Each corresponding SL sequences is also colored according to the 27 SLs. The colors of the 27 SLs indicate the secondary structure that each SL describes. [a, A, V, W]-SLs that are primarily found in the α-helix are colored in red, and [L, M, N, T, X]-SLs that are primarily found in the β-strand are colored in green. In the middle of the figure, HMM-SA is presented: the geometry of its 27 SLs (in right) and the α-RMSD between all SL-pairs. SLs are sorted left-right, top-bottom by increasing stretches.
(TIFF)

S3 Fig Sequence variability of 107 PDB files corresponding to UniProt ID P00749. MSA computed using the 107 PDB files corresponding to UniProt ID P00749 (step 2 output of SA-conf). and Clustalw software. Rows represent the 184 protein chains, and columns correspond to the 387 MSA positions. The AAs of all sequences are colored according to the 20 AA types.
(TIF)

S4 Fig Sequence and structural diversity of different sets extracted from the 78 PDB files corresponding to UniProt ID P04637 or P02340. (A-B) MSA graphics (SA-conf Step 2 output: AA_alignment.pdf). (A) MSA computed using the 203 chains extracted from the 78 PDB files corresponding to the UniProt ID P04637 or P02340. (B) MSA computed using the P53 set. In these two graphics, the AA sequences of chains are presented in rows, and the positions are presented in columns. Each aligned position is colored according to the 20 AA types as shown in (A). (C-D) MSLA graphics (SA-conf Step 3 output: SL_alignment.pdf) computed using the P53 set (C) and the P53-NMR set (D). In the MSLA, the SL series of the MTC are presented in rows, and the MSA positions are presented in columns. Positions are colored according to the 27 SLs as shown in (C). The colors of the 27 SLs indicate the secondary structure that each SL describes. [a, A, V, W]-SLs that are primarily found in the α-helix are colored in red and [L, M, N, T, X]-SLs that are primarily found in the β-strand are colored in green.
(PDF)

S5 Fig Visualization of the sequence and structural variability of the P53 set. (A) MSA (SA-conf step 2 output named AA_alignment.pdf) obtained using the set of 78 p53 DBD domains. The 78 aligned AA sequences are presented in rows, and the 241 MSA positions are presented in columns. Each position is colored according to the 20 AA types. (B) MSLA (SA-conf step 3 output named SL_alignment.pdf) computed using the 78 p53 DBD domains. The 78 aligned SL sequences are presented in rows, and the 241 MSA positions are presented in columns and are colored according to the 27 SLs. Colors of the 27 SLs indicate the secondary structure that each SL describes. [a, A, V, W]-SLs primarily found in the α-helix are colored in red, and [L, M, N, T, X]-SLs primarily found in the β-strand are colored in green.
(TIFF)

S6 Fig Structural diversity of PR1 set. MSLA graphics (SA-conf Step 3 output: SL_alignment.pdf) computed using the PR1 set. In the MSLA, the SL series of the MTC are presented in rows, and the MSA positions are presented in columns. Positions are colored according to the 27 SLs. The colors of the 27 SLs indicate the secondary structure that each SL describes. [a, A, V, W]-SLs that are primarily found in the α-helix are colored in red and [L, M, N, T, X]-SLs that are primarily found in the β-strand are colored in green.
(TIFF)

S7 Fig Sequence and structural analysis of the PR1 set. Representation of the *neq*_*AA*_ (bottom graph) and *neq*_*SL*_ (top graph) values along the 99 MSA positions in the PR1 set (Step 4 output: Neq_graph.pdf). Bars presenting *neq*_*SL*_ values are colored according to their secondary structure status: red presents the positions in which all chains have an α-helix conformation, magenta presents the positions in which all chains have a β-strand conformation, gray presents the aligned positions in which all chains have a loop conformation, and purple presents the aligned positions where secondary structure changes occur. In this figure, we added blue rectangles to localize the 8 variable regions highlighted during the PR1 set analysis: R1_PR1_ (positions 3–6), R2_PR1_ (positions 9–13), R3_PR1_ (positions 37–44), R4_PR1_ (positions 46–51), R5_PR1_ (positions 57–60), R6_PR1_ (positions 64–67), R7_PR1_ (positions 86–93) and R8_PR1_ (positions 95–98).
(TIFF)

S8 Fig Sequence and structural analysis of the PR1-NMR set. Representation of the *neq*_*AA*_ (bottom graph) and *neq*_*SL*_ (top graph) values along the 99 MSA positions in the PR1 set (Step 4 output: Neq_graph.pdf). Bars presenting *neq*_*SL*_ values are colored according to their secondary structure status: red presents the positions in which all chains have an α-helix conformation, magenta presents the positions in which all chains have a β-strand conformation, gray presents the aligned positions in which all chains have a loop conformation, and purple presents the aligned positions where secondary structure changes occur. In this figure, we added blue rectangles to localize the 8 variable regions highlighted during the PR1 set analysis: R1_PR1-NMR_ (positions 3–15), R2_PR1-NMR_ (positions 34–44), R3_PR1-NMR_ (positions 46–49), R4_PR1-NMR_ (positions 57–61), R5_PR1-NMR_ (positions 63–67), R6_PR1-NMR_ (positions 88–93) and R7_PR1_ (positions 96–98).
(TIFF)

S9 Fig Sequence and structural analysis of the P53-NMR set. Representation of the *neq*_*AA*_ (bottom graph) and *neq*_*SL*_ (top graph) values along the 241 MSA positions in the P53-NMR dataset (Step 4 output: Neq_graph.pdf). Bars presenting *neq*_*SL*_ values are colored according to their secondary structure status: red presents the positions in which all chains have an α-helix conformation, magenta presents the positions in which all chains have a β-strand conformation, gray presents the aligned positions in which all chains have a loop conformation, and purple presents the aligned positions where secondary structure changes occur. In this figure, we added blue rectangles to localize the 7 variable regions highlighted during the P53-NMR set analysis: R1_P53-NMR_ (positions 3–16), R2_P53-NMR_ (positions 18–35), R3_P53-NMR_ (positions 70–101), R4_P53-NMR_ (positions 124–144), R5_P53-NMR_ (positions 148–154), R6_P53-NMR_ (positions 168–174), and R7_P53-NMR_ (positions 193–203).
(TIFF)

S1 Table Comparison between structural changes captured by *neq*_*SL*_ parameter and those captured by secondary structure information. Secondary structures were defined using HMM-SA definition (Regad et al., 2016) and were provided by SA-conf.
(XLSX)

S2 Table Characterization of the inhibitor-binding site of the uPA catalytic domain. uPA catalytic domain residues involved in the pocket binding of small molecules were extracted using PockDrug server with PDB ID 3I6G as input. The two first columns correspond to the position of pocket residues identified in the input PDB file (column 1) and in MSA (column 2) positions. The third column contains information on the involvement of pocket residues in the interaction with the co-crystallized ligand (478, het atom code). Hydrophobic and hydrogen bonds were extracted using LigPlot software and PDB file 3I6G (uPA catalytic domain complexed with the 478 ligand). The fourth and fifth columns indicate the *neq*_*AA*_ and *neq*_*SL*_ values for each pocket residue. The last column displays the named structural variable region where the pocket residues are located.
(PDF)

S3 Table Comparison between structural changes captured by SA-conf and PSSweb (Gaillard et al., 2013; 2016). The table presents the Pearson coefficient correlation (r) computed between *neq*_*SL*_ values provides by SA-conf and backbone *rmsf* value provided by PSSweb of each MSA position for different MTC subsets.
(XLSX)

## Abbreviations

3D: three-dimensional
AA: amino acid
DBD: DNA-binding domain of p53
HMM-SA: Hidden Markov Model—Structural Alphabet
MSA: multiple sequence alignment
MSLA: multiple structural letter alignment
MTC: multiple target conformations
NMR: nuclear magnetic resonance
PR1: protease of the immunodeficiency virus type 1
SA: structural alphabet
SL: structural letter
uPA: urokinase-type plasminogen activator
