# Supplementary figures and images for: Exploring the potential of a structural alphabet-based tool for mining multiple target conformations and target flexibility insight
Source: PLoS One. 2017 Aug 17;12(8):e0182972. doi: 10.1371/journal.pone.0182972 (PMC5560695; doi:10.1371/journal.pone.0182972)

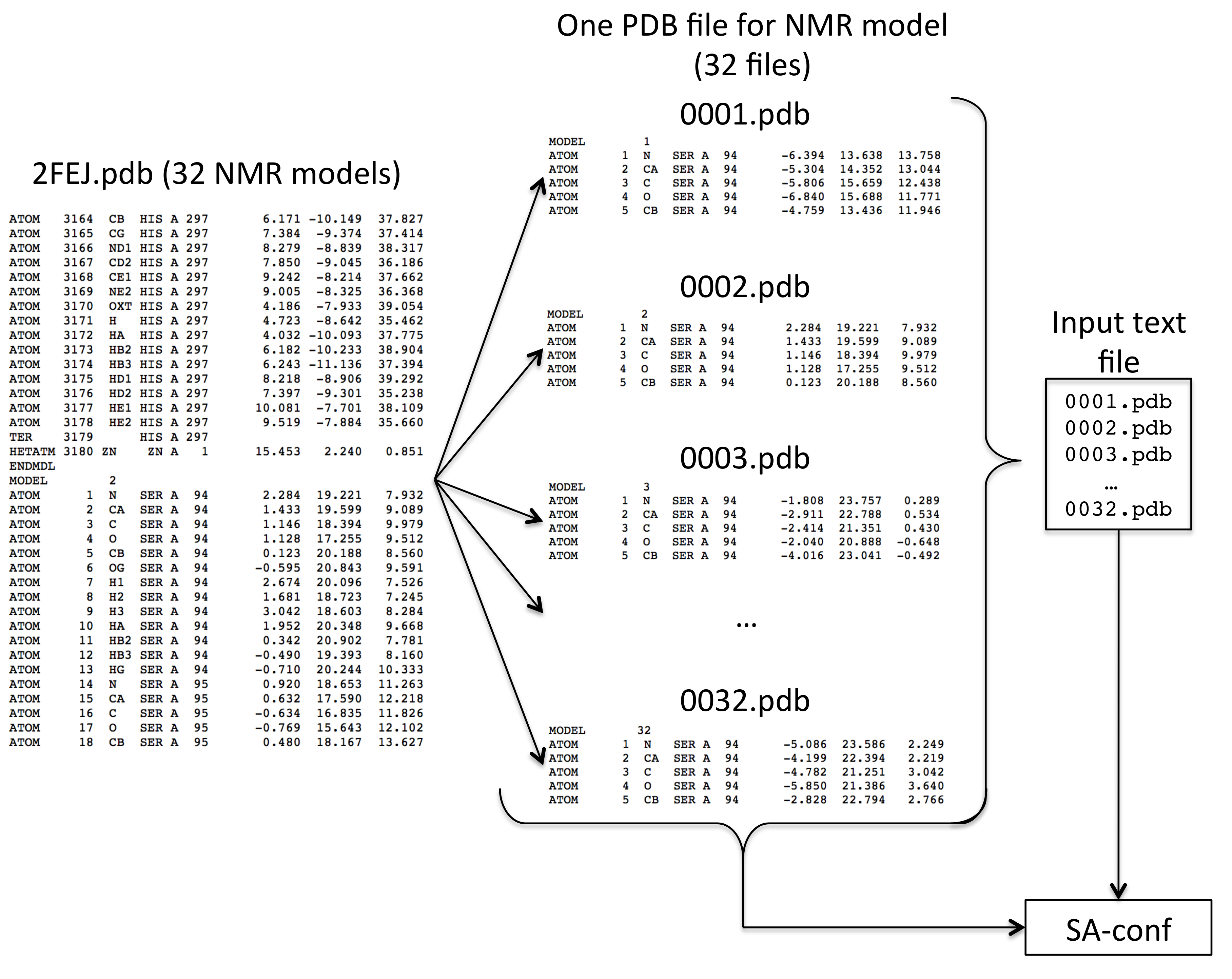

Supplement: S1 Fig — 3D coordinates of each NMR model are extracted from the PDB files and stored in a text file in PDB format, which the name correspond to an artificial PDB ID (four characters + “.pdb”). The artificial name of each created PDB file are stored in a text file. This text file and all created PDB files will be the input of the SA-conf software, as illustrated for the 2FEJ PDB file. (TIFF) [file pone.0182972.s001.tiff]

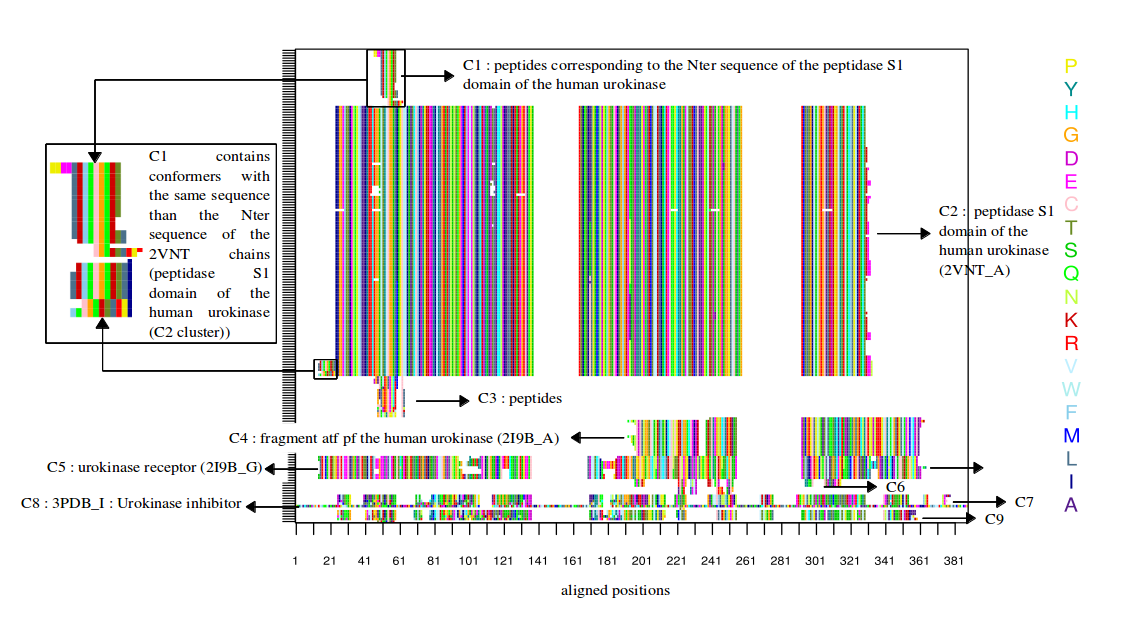

Supplement: S3 Fig — MSA computed using the 107 PDB files corresponding to UniProt ID P00749 (step 2 output of SA-conf). and Clustalw software. Rows represent the 184 protein chains, and columns correspond to the 387 MSA positions. The AAs of all sequences are colored according to the 20 AA types. (TIF) [file pone.0182972.s003.tif]

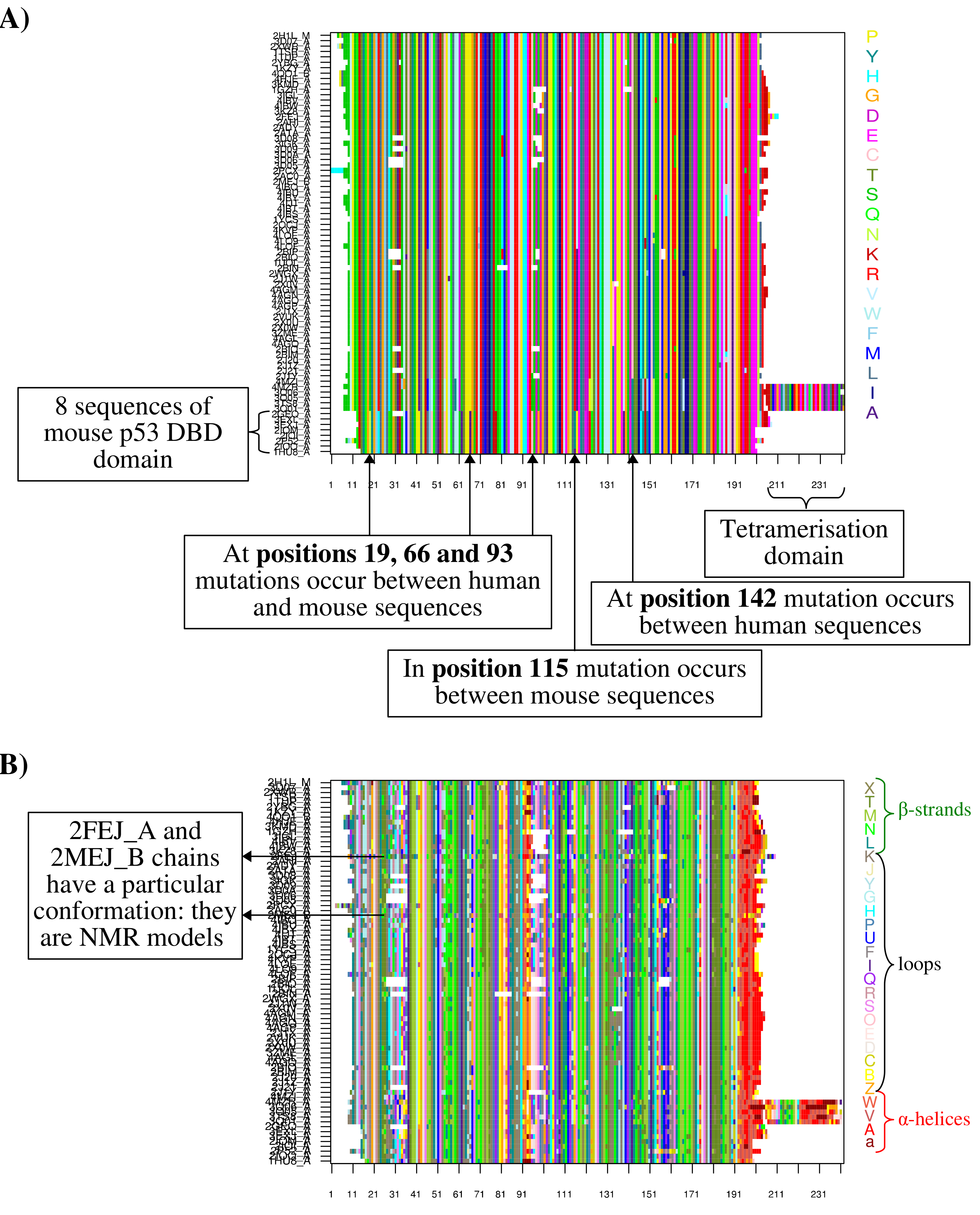

Supplement: S5 Fig — (A) MSA (SA-conf step 2 output named AA_alignment.pdf) obtained using the set of 78 p53 DBD domains. The 78 aligned AA sequences are presented in rows, and the 241 MSA positions are presented in columns. Each position is colored according to the 20 AA types. (B) MSLA (SA-conf step 3 output named SL_alignment.pdf) computed using the 78 p53 DBD domains. The 78 aligned SL sequences are presented in rows, and the 241 MSA positions are presented in columns and are colored according to the 27 SLs. Colors of the 27 SLs indicate the secondary structure that each SL describes. [a, A, V, W]-SLs primarily found in the α-helix are colored in red, and [L, M, N, T, X]-SLs primarily found in the β-strand are colored in green. (TIFF) [file pone.0182972.s005.tiff]

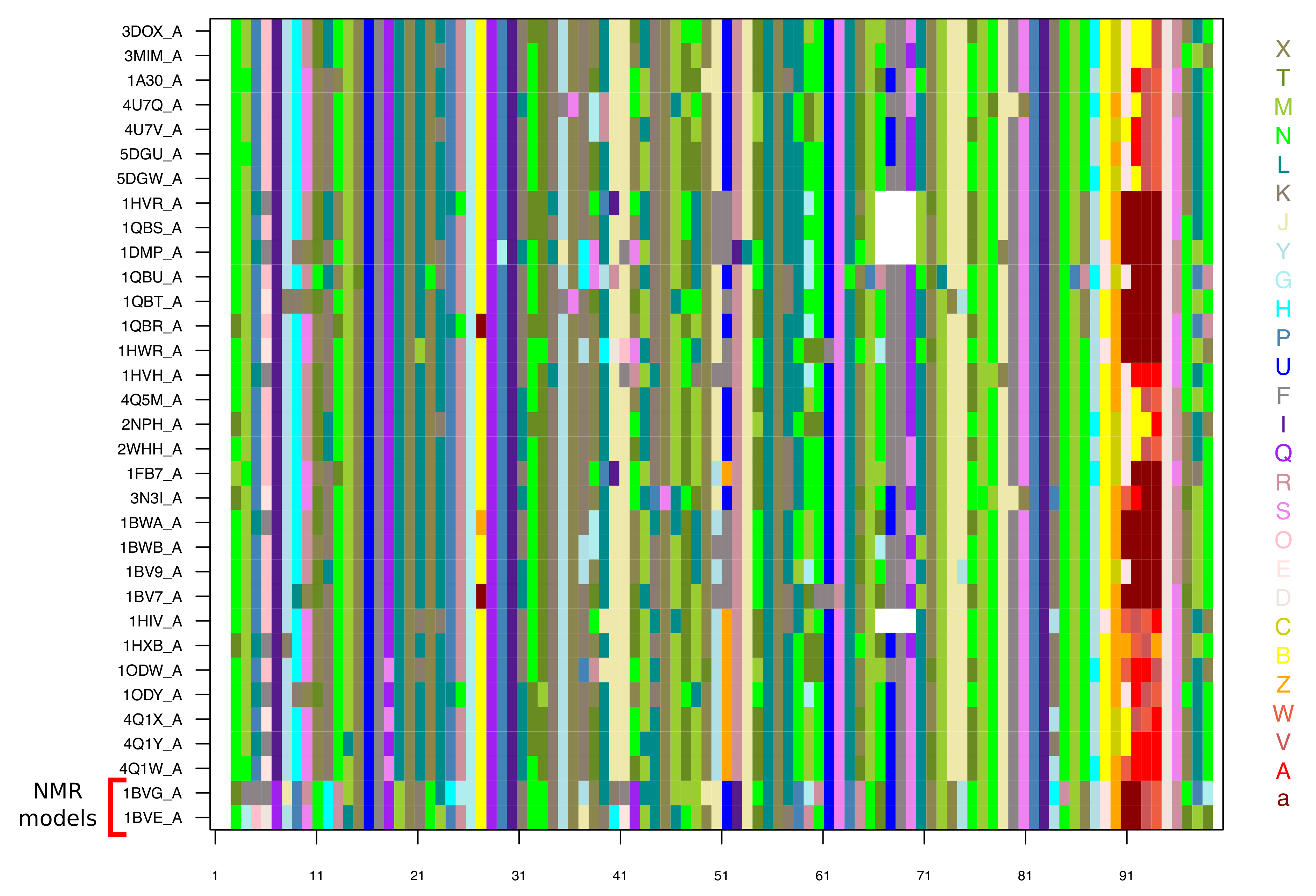

Supplement: S6 Fig — MSLA graphics (SA-conf Step 3 output: SL_alignment.pdf) computed using the PR1 set. In the MSLA, the SL series of the MTC are presented in rows, and the MSA positions are presented in columns. Positions are colored according to the 27 SLs. The colors of the 27 SLs indicate the secondary structure that each SL describes. [a, A, V, W]-SLs that are primarily found in the α-helix are colored in red and [L, M, N, T, X]-SLs that are primarily found in the β-strand are colored in green. (TIFF) [file pone.0182972.s006.tiff]

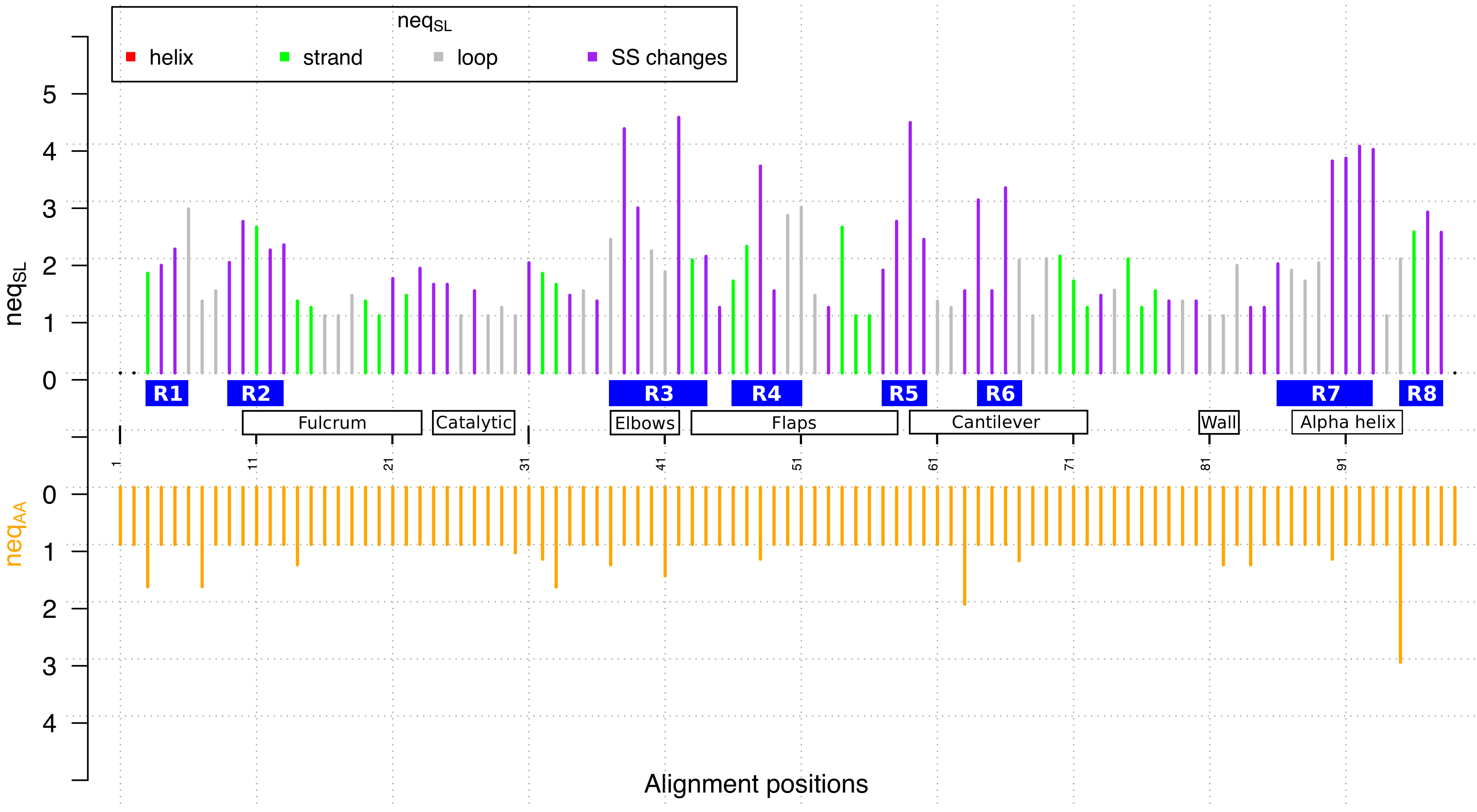

Supplement: S7 Fig — Representation of the neqAA (bottom graph) and neqSL (top graph) values along the 99 MSA positions in the PR1 set (Step 4 output: Neq_graph.pdf). Bars presenting neqSL values are colored according to their secondary structure status: red presents the positions in which all chains have an α-helix conformation, magenta presents the positions in which all chains have a β-strand conformation, gray presents the aligned positions in which all chains have a loop conformation, and purple presents the aligned positions where secondary structure changes occur. In this figure, we added blue rectangles to localize the 8 variable regions highlighted during the PR1 set analysis: R1PR1 (positions 3–6), R2PR1 (positions 9–13), R3PR1 (positions 37–44), R4PR1 (positions 46–51), R5PR1 (positions 57–60), R6PR1 (positions 64–67), R7PR1 (positions 86–93) and R8PR1 (positions 95–98). (TIFF) [file pone.0182972.s007.tiff]

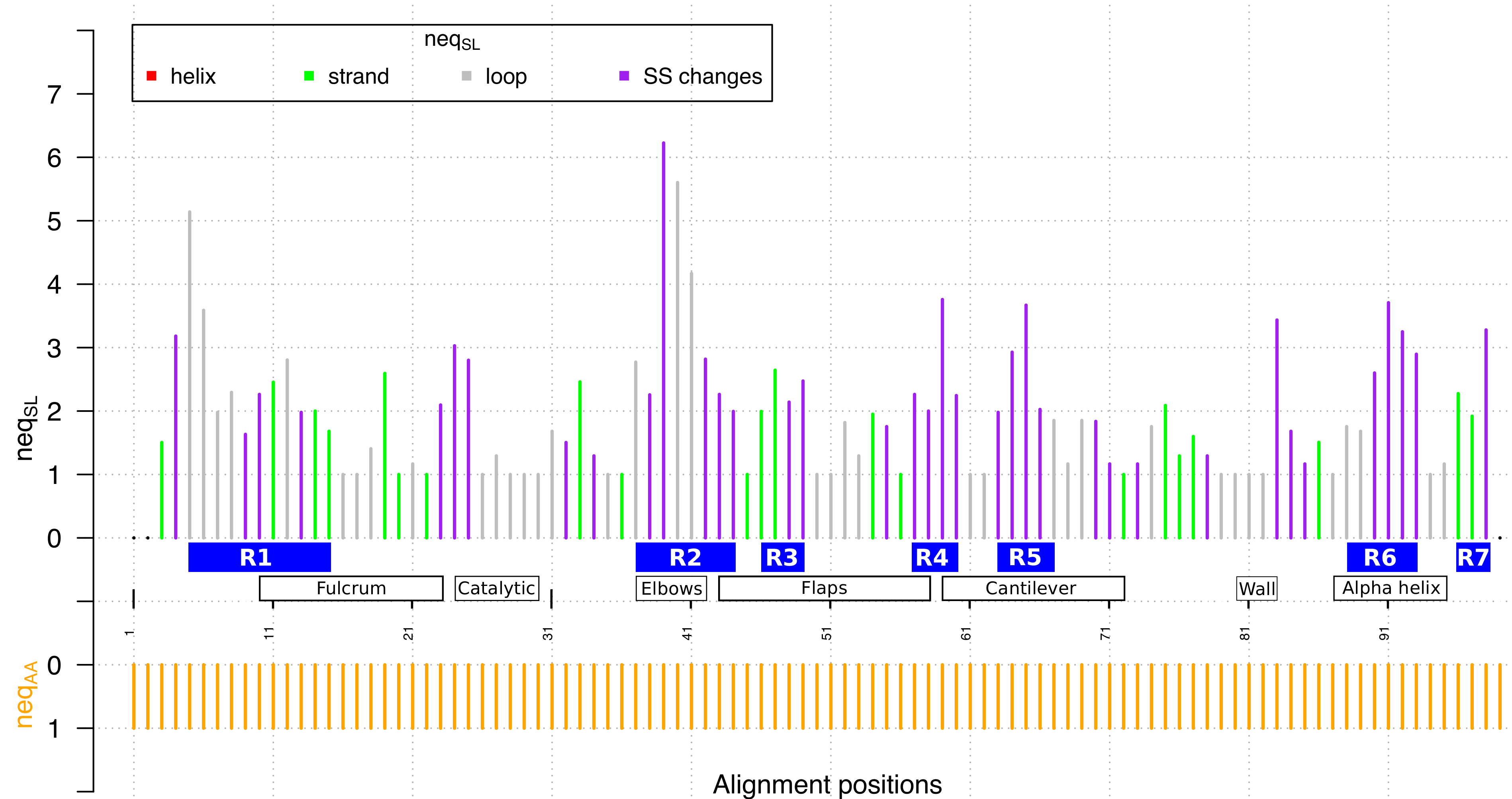

Supplement: S8 Fig — Representation of the neqAA (bottom graph) and neqSL (top graph) values along the 99 MSA positions in the PR1 set (Step 4 output: Neq_graph.pdf). Bars presenting neqSL values are colored according to their secondary structure status: red presents the positions in which all chains have an α-helix conformation, magenta presents the positions in which all chains have a β-strand conformation, gray presents the aligned positions in which all chains have a loop conformation, and purple presents the aligned positions where secondary structure changes occur. In this figure, we added blue rectangles to localize the 8 variable regions highlighted during the PR1 set analysis: R1PR1-NMR (positions 3–15), R2PR1-NMR (positions 34–44), R3PR1-NMR (positions 46–49), R4PR1-NMR (positions 57–61), R5PR1-NMR (positions 63–67), R6PR1-NMR (positions 88–93) and R7PR1 (positions 96–98). (TIFF) [file pone.0182972.s008.tiff]

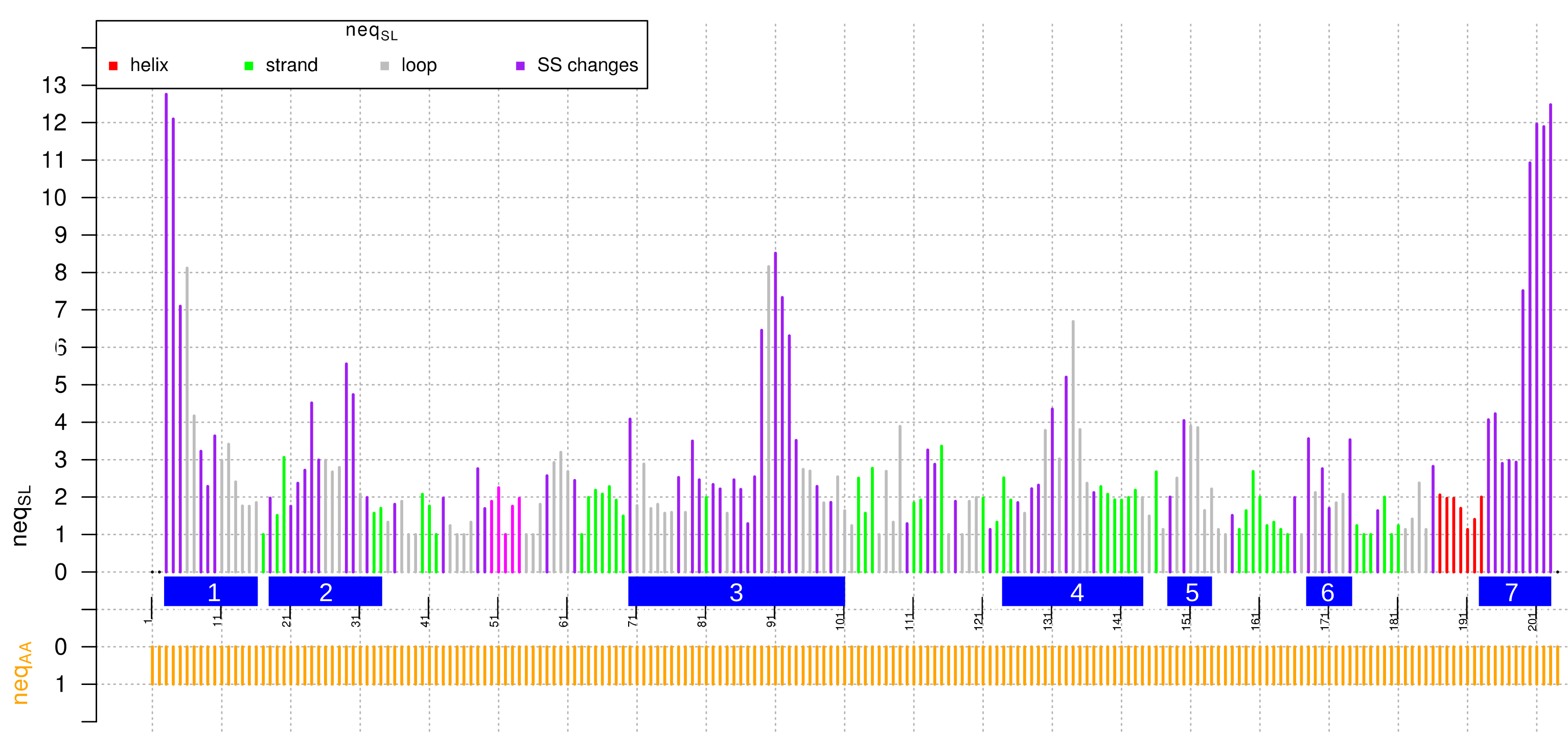

Supplement: S9 Fig — Representation of the neqAA (bottom graph) and neqSL (top graph) values along the 241 MSA positions in the P53-NMR dataset (Step 4 output: Neq_graph.pdf). Bars presenting neqSL values are colored according to their secondary structure status: red presents the positions in which all chains have an α-helix conformation, magenta presents the positions in which all chains have a β-strand conformation, gray presents the aligned positions in which all chains have a loop conformation, and purple presents the aligned positions where secondary structure changes occur. In this figure, we added blue rectangles to localize the 7 variable regions highlighted during the P53-NMR set analysis: R1P53-NMR (positions 3–16), R2P53-NMR (positions 18–35), R3P53-NMR (positions 70–101), R4P53-NMR (positions 124–144), R5P53-NMR (positions 148–154), R6P53-NMR (positions 168–174), and R7P53-NMR (positions 193–203). (TIFF) [file pone.0182972.s009.tiff]
