# Supplementary material for: Exploring the potential of a structural alphabet-based tool for mining multiple target conformations and target flexibility insight
Source: PLoS One. 2017 Aug 17;12(8):e0182972. doi: 10.1371/journal.pone.0182972 (PMC5560695; doi:10.1371/journal.pone.0182972)
Supplement: S2 Table — uPA catalytic domain residues involved in the pocket binding of small molecules were extracted using PockDrug server with PDB ID 3I6G as input. The two first columns correspond to the position of pocket residues identified in the input PDB file (column 1) and in MSA (column 2) positions. The third column contains information on the involvement of pocket residues in the interaction with the co-crystallized ligand (478, het atom code). Hydrophobic and hydrogen bonds were extracted using LigPlot software and PDB file 3I6G (uPA catalytic domain complexed with the 478 ligand). The fourth and fifth columns indicate the neqAA and neqSL values for each pocket residue. The last column displays the named structural variable region where the pocket residues are located. (PDF) [file pone.0182972.s011.pdf]

PDB positions	MSA positions	Bond types	neq_{AA}	neq_{SL}	Structural variable region
40	41		1	1.31	
41	***42***	***Hydrophobic***	***1***	***1.74***	***R1***
42	***43***	***Hydrophobic***	***1***	***2.64***	***R1***
57	***58***	Hydrogen	***1***	***1.41***	
58	59		1	1.5	
96	101	Hydrophobic	1	1.71	R3
97	102		1.06	2.73	R3
97A	103		1	2.3	R3
97B	104	***Hydrophobic***	1	2.35	R3
98	105	***Hydrophobic***	1	1.59	R3
99	106	***Hydrophobic***	1	1.52	R3
102	109	***Hydrogen***	1	1.1	
189	204	***Hydrogen***	1	**1.06**	
190	205	***Hydrogen***	1.14	1.36	
191	206	***Hydrophobic***	1	***1***	
192	207	***Hydrophobic***	1	1	
193	208	Hydrogen	1	1.1	
194	209		1	1.06	
195	210	Hydrogen	1.14	1.27	
213	228	Hydrophobic	1	2.07	
215	230	***Hydrophobic***	1	1.37	
216	231	***Hydrophobic***	1	1.97	R8
217	232		1	2.23	R8
218	233	***Hydrogen***	1	2.19	R8
220	***234***	***Hydrophobic***	1	1.9	R8
221	235		1	1.06	
226	241		1	1.06	
