# Supplementary material for: Exploring the potential of a structural alphabet-based tool for mining multiple target conformations and target flexibility insight
Source: PLoS One. 2017 Aug 17;12(8):e0182972. doi: 10.1371/journal.pone.0182972 (PMC5560695; doi:10.1371/journal.pone.0182972)
Supplement: S4 Fig — (A-B) MSA graphics (SA-conf Step 2 output: AA_alignment.pdf). (A) MSA computed using the 203 chains extracted from the 78 PDB files corresponding to the UniProt ID P04637 or P02340. (B) MSA computed using the P53 set. In these two graphics, the AA sequences of chains are presented in rows, and the positions are presented in columns. Each aligned position is colored according to the 20 AA types as shown in (A). (C-D) MSLA graphics (SA-conf Step 3 output: SL_alignment.pdf) computed using the P53 set (C) and the P53-NMR set (D). In the MSLA, the SL series of the MTC are presented in rows, and the MSA positions are presented in columns. Positions are colored according to the 27 SLs as shown in (C). The colors of the 27 SLs indicate the secondary structure that each SL describes. [a, A, V, W]-SLs that are primarily found in the α-helix are colored in red and [L, M, N, T, X]-SLs that are primarily found in the β-strand are colored in green. (PDF) [file pone.0182972.s004.pdf]

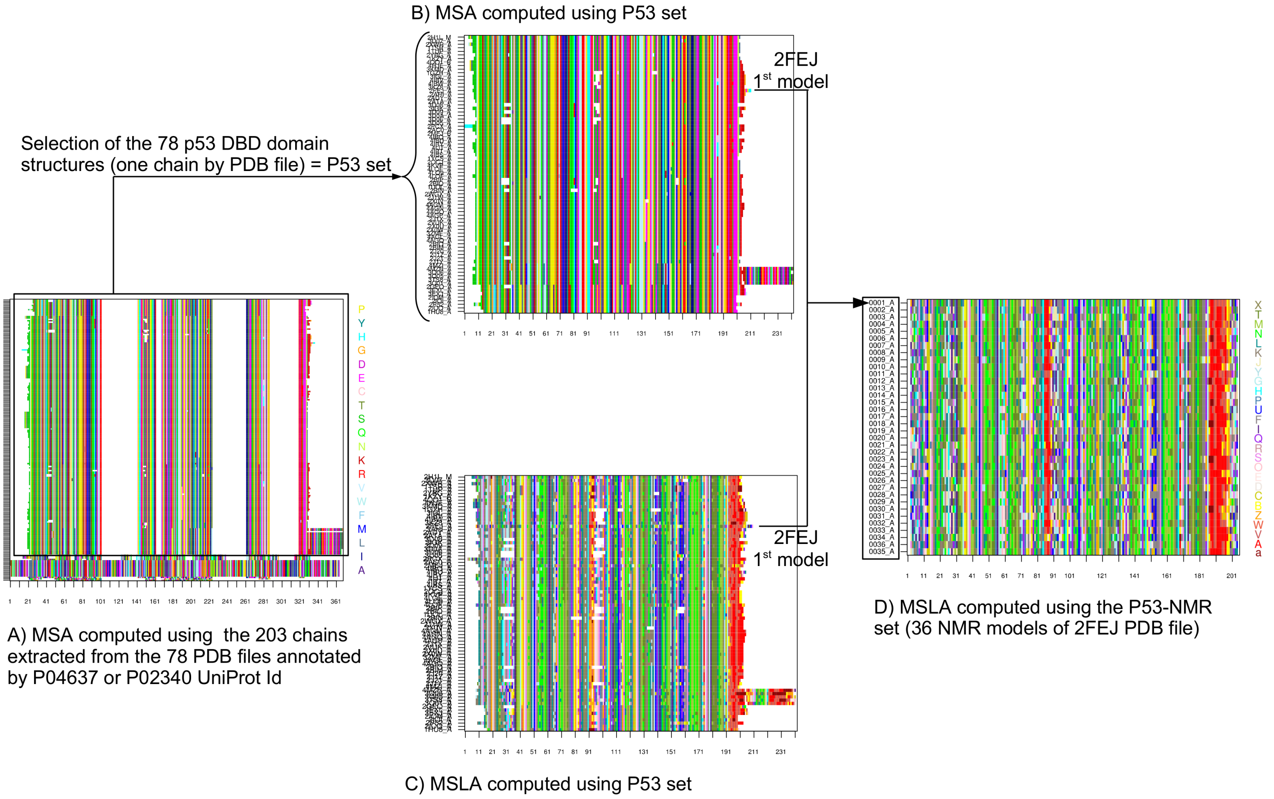

B) MSA computed using P53 set
2FEJ
1st model
Selection of the 78 p53 DBD domain structures (one chain by PDB file) = P53 set
A) MSA computed using the 203 chains extracted from the 78 PDB files annotated by P04637 or P02340 UniProt Id
C) MSLA computed using P53 set
D) MSLA computed using the P53-NMR set (36 NMR models of 2FEJ PDB file)
